# Supplementary material for: Intrinsic TGF-β signaling attenuates proximal tubule mitochondrial injury and inflammation in chronic kidney disease
Source: Nat Commun. 2023 Jun 3;14:3236. doi: 10.1038/s41467-023-39050-y (PMC10239443; doi:10.1038/s41467-023-39050-y)
Supplement: Supplementary file 1 — Supplementary information [file 41467_2023_39050_MOESM1_ESM.pdf]

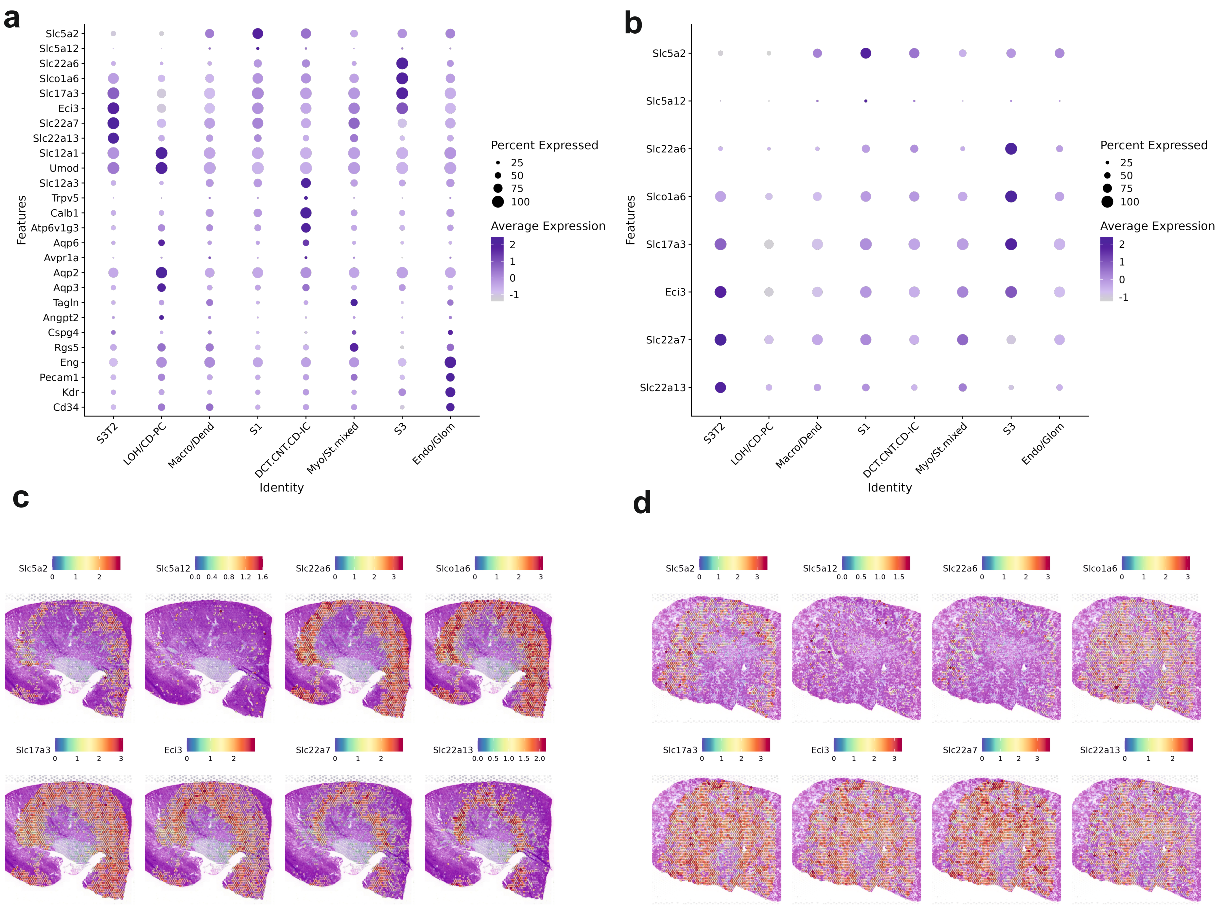

Supplementary Fig. 1: (a,b) Dotplots depicting cell type markers and (b) selected PT markers (S1,S3 and S3T2). (c,d) Representative spatial featplot of PT markers resolved on uninjured (c) and injured (d) kidneys showing specific spatial localization of PT cells in uninjured and impairement under injury.

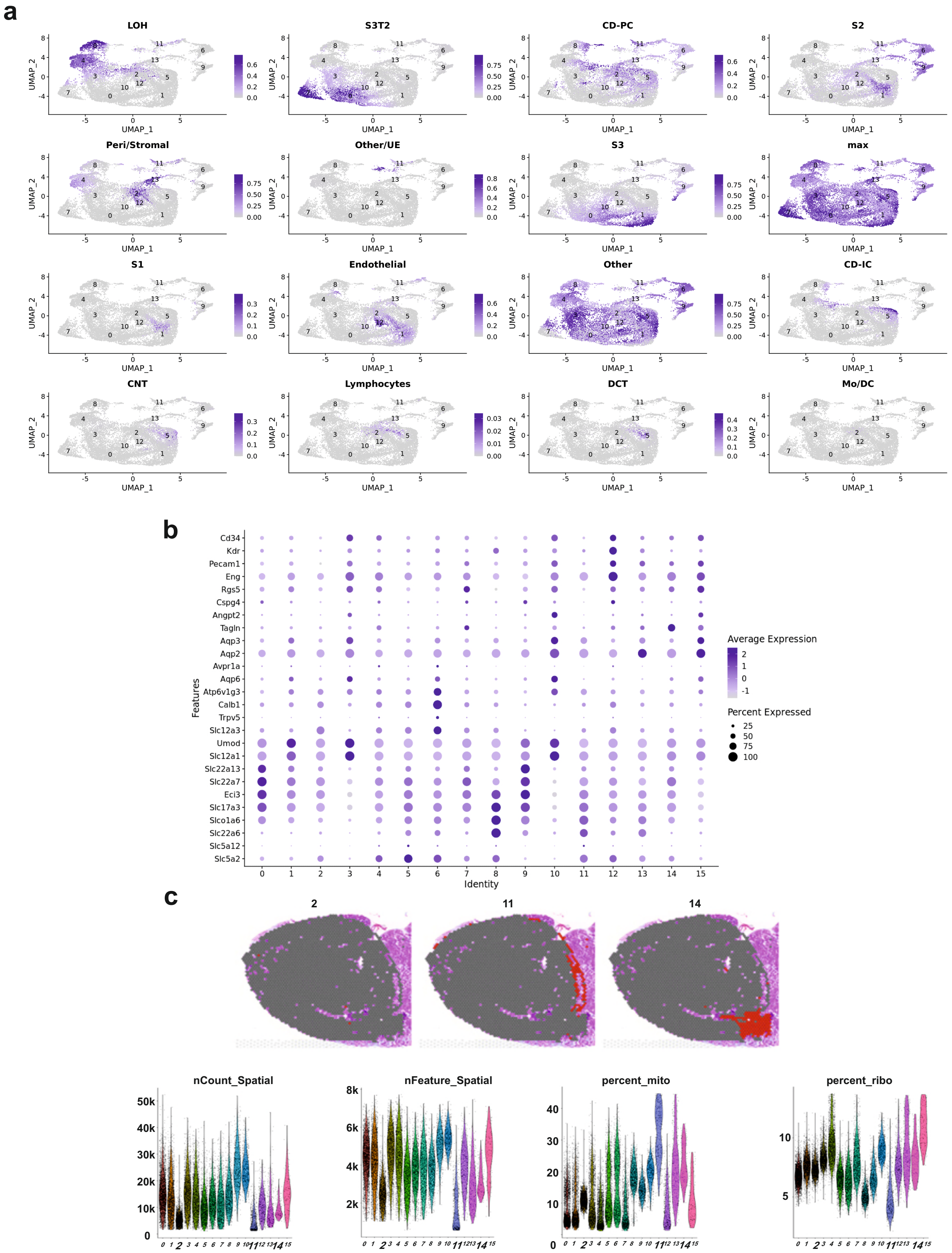

Supplementary Fig. 2: Spatial transcriptomics cluster annotation and cell type identification. (a, b) Probabilistic transfer of single-cell data to spatial transcriptomics, showing cluster prediction scores and specific marker expression features per cluster. (c) Representative cluster resolution on H&E stained kidney images and multimetric quality control analysis used for data curating and removal of low quality clusters (e.g. 2, 11 and 14) preformed on all spatial transcriptomics datasets.

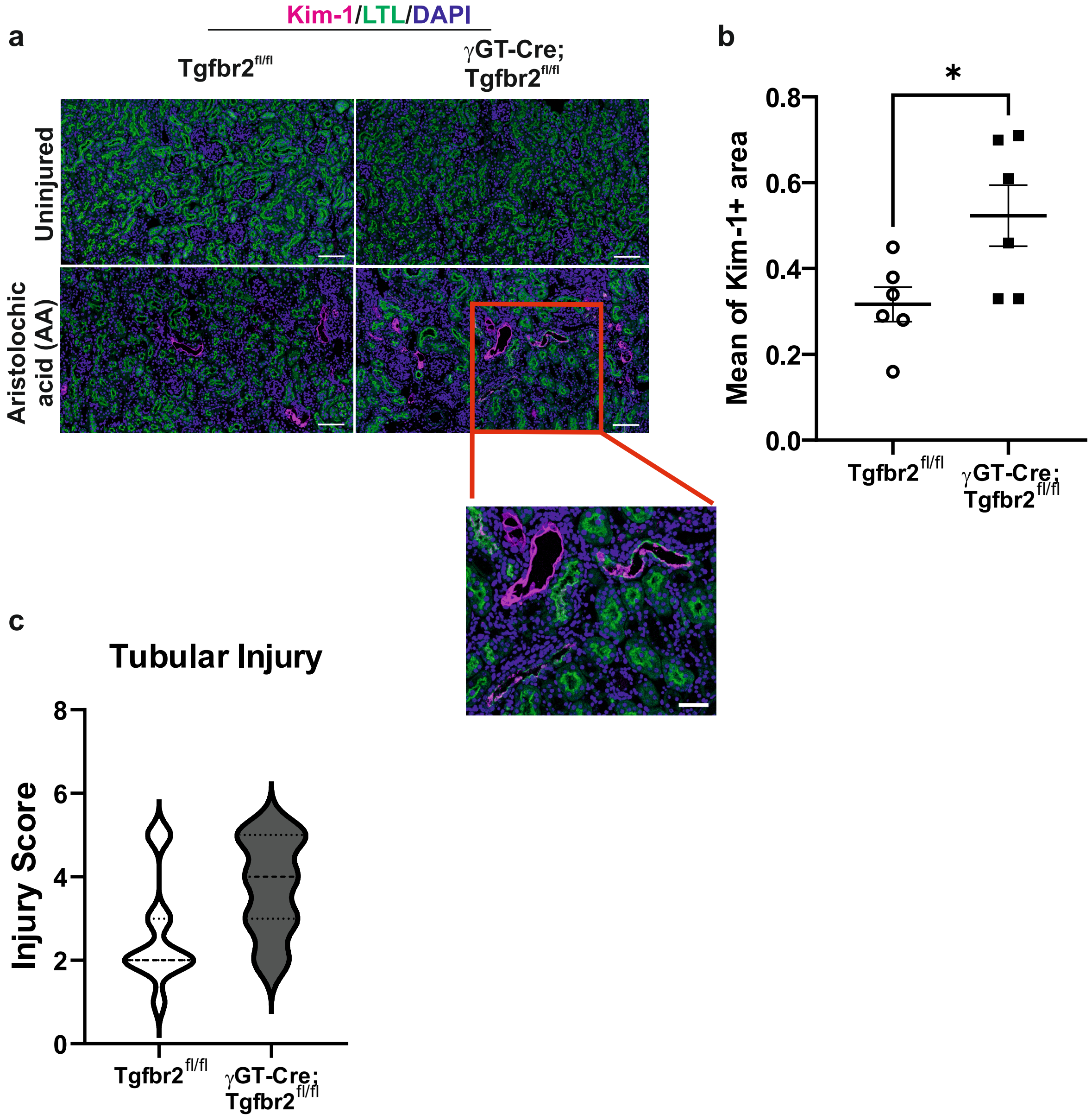

Supplementary Fig. 3: (a, b) Representative immunofluorescence images and quantification of Kim-1 protein expression showing increased injured proximal tubules in the renal cortices of γGT-Cre;Tgfbr2$^{fl/fl}$ mice compared to their Tgfbr2$^{fl/fl}$ littermates 3 weeks after AA injury; n=6 (Tgfbr2$^{fl/fl}$) and 6 (γGT-Cre;Tgfbr2$^{fl/fl}$) mice, *p*= 0.0302. Scale bar=50μm. (c) H&E injury quantification score showing increased tubular injury in γGT-Cre;Tgfbr2$^{fl/fl}$ mice compared to their Tgfbr2$^{fl/fl}$ littermates 6 weeks after AA injury; n=11 (Tgfbr2$^{fl/fl}$) and 12 (γGT-Cre;Tgfbr2$^{fl/fl}$) mice, *p*= 0.0389. Data are presented as mean values ±SEM. Statistical significance was determined by unpaired Student's *t* test (two groups) with p<0.05 considered statistically significant. The dots represents the number of animals per group (b). * represents p<0.05. Source data are provided as a Source Data file.

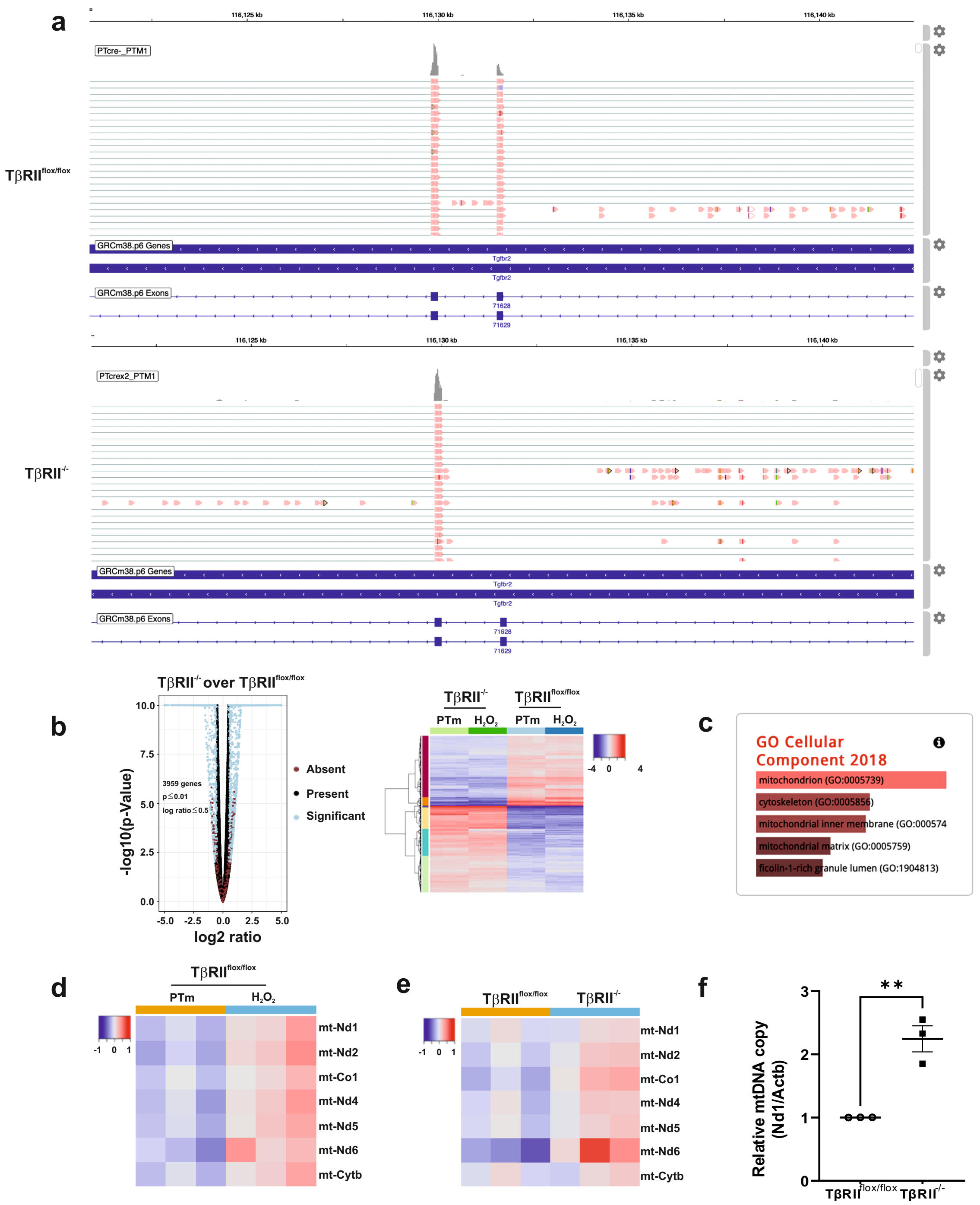

Supplementary Fig. 4: Proximal tubule TβRII deletion affects mitochondrial homeostasis. (a) Integrative Genomics Viewer images of sequencing showing the deletion of TβRII exon 2 in TβRII$^{-/-}$ PT cells. (b) Volcano plot and heatmap of an RNAseq analysis of PT cells treated or not with H_2O_2 showing 3359 genes deregulated genes (p<0.01 and fc≥ 1.5) in TβRII$^{-/-}$ compared to TβRII$^{flox/flox}$ PT cells. (c) Overrepresentation analysis using EnrichR revealed mitochondrion as the top affected cellular component in TβRII$^{-/-}$ PT cells as compared to TβRII$^{flox/flox}$ PT cells. (d) Heatmap of TβRII$^{flox/flox}$ PT cells treated or not with H_2O_2 showing oxidative stress induction of mt-genome encoded mRNAs of electron transport chain subunits. (e) Heatmap of PT cells showing

basal induction of mt-genome encoded electron transport chain subunit mRNA in TβRII$^{-/-}$ PT cells compared to TβRII$^{flox/flox}$ PT cells. (f) Mitochondrial DNA synthesis measured by RT-qPCR using the mt-genome encoded gene (Nd1) and normalized to the nuclear genome encoded gene (β-actin) showing increased mitochondrial biogenesis in TβRII$^{-/-}$ PT cells (n=3 independent experiments, *p*= 0.0038). Data are presented as mean values ±SEM. Statistical significance was determined by unpaired Student's *t* tests. **p represents <0.01. Source data are provided as a Source Data file.

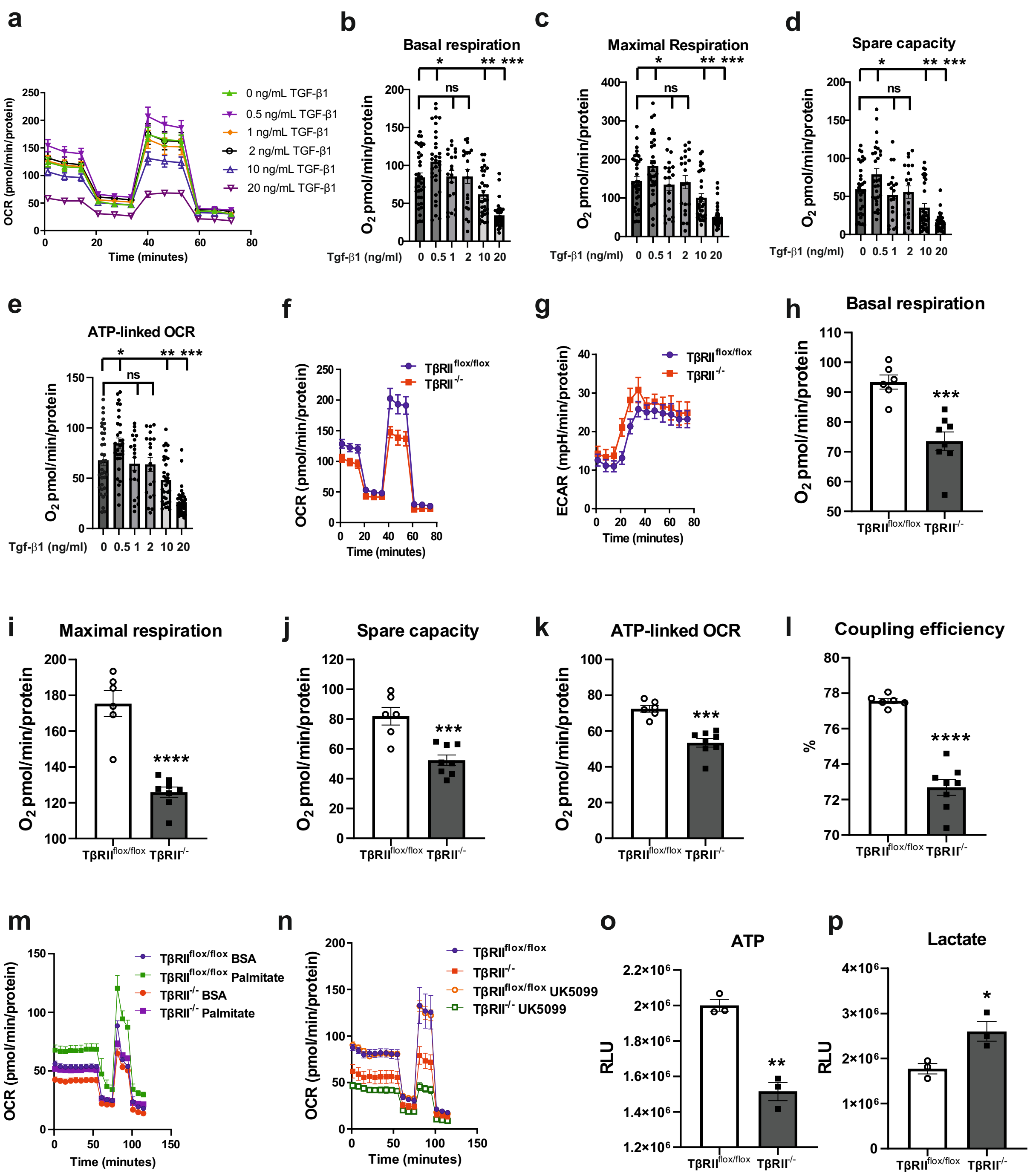

Supplementary Fig. 5: Proximal tubule TβRII deletion disrupts mitochondrial function and metabolism in vitro. (a-e) Seahorse analyses showing dose-dependent effect of TGF-β on TβRII$^{flox/flox}$ PT cell respiration (basal and maximal) and ATP production through OXPHOS (spare capacity and ATP-link OCR) (n=3 independent experiments). (f-l) Seahorse analyses showing decreased respiration (basal p= 0.0005 and p<0.0001 maximal), spare capacity (p= 0.0007) and ATP-linked OCR (p< 0.0001) and slightly increased extracellular acidification rate (ECAR) in TβRII$^{-/-}$ compared to TβRII$^{flox/flox}$ PT cells. Seahorse analyses of coupling efficiency (p<0.0001)

showing a decrease in $T\beta RII^{-/-}$ compared to $T\beta RII^{flox/flox}$ PT cells (n=3 independent experiments). (m) Representative graph of seahorse analysis of fatty acid oxidation showing decreased residual ability to metabolize fatty acid in $T\beta RII^{-/-}$ compared to $T\beta RII^{flox/flox}$ PT cells (n= 3 independent biological replicates). (n) Representative graph of seahorse analyses of substrate dependency indicating increased glycolytic dependency in $T\beta RII^{-/-}$ compared to $T\beta RII^{flox/flox}$ PT cells (n= 3 independent biological replicates). (o) Bioluminescence measurement of ATP production showing decreased production in $T\beta RII^{-/-}$ compared to $T\beta RII^{flox/flox}$ PT cells (n=3 independent experiments, *p*= 0.0014). (p) Bioluminescence measurement of lactate production showing increased production in $T\beta RII^{-/-}$ compared to $T\beta RII^{flox/flox}$ PT cells (n=3 independent experiments *p*= 0.0281). Data are presented as mean values ±SEM. Statistical significance was determined by unpaired Student's *t* test (two groups) with $p<0.05$ considered statistically significant.* represents $p<0.05$; ** represents $p<0.01$; *** represents $p<0.001$; **** represents $p<0.0001$. Source data are provided as a Source Data file.

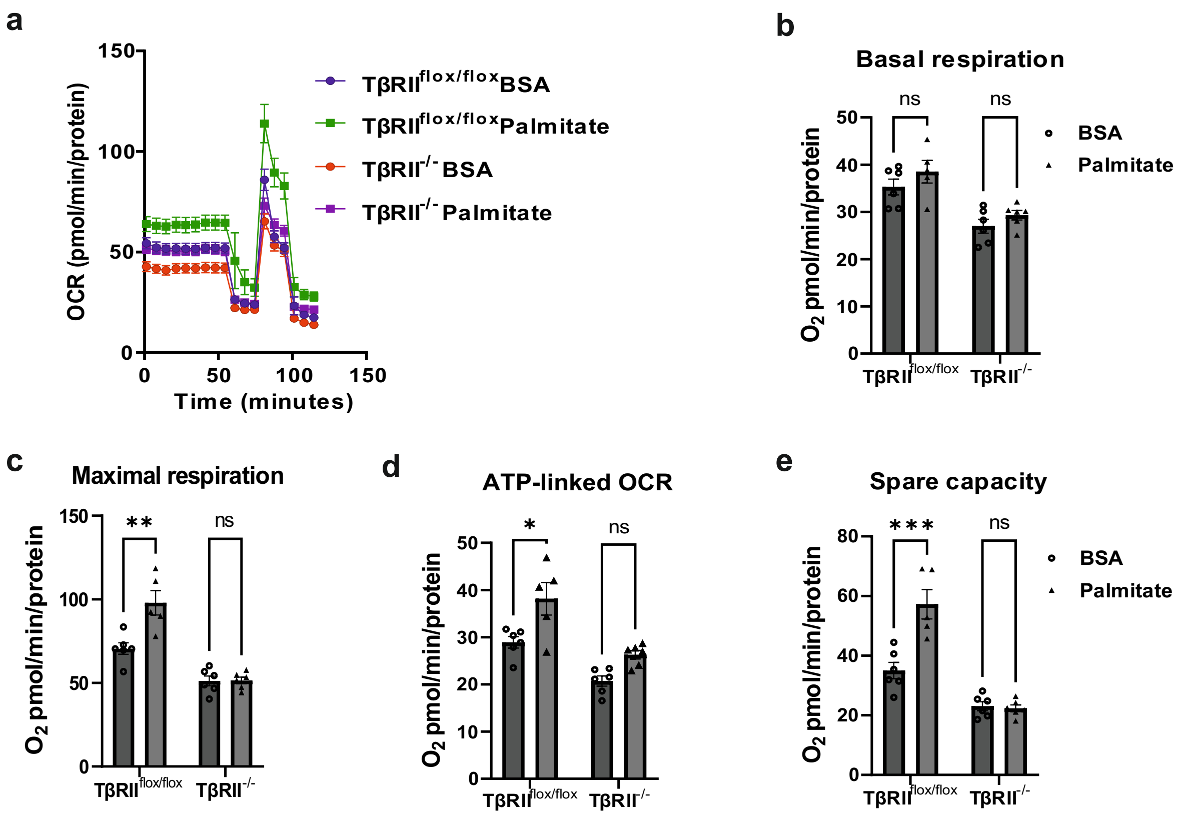

Supplementary Fig. 6: Supplementary Fig. 2: Seahorse analysis of fatty acid metabolism indicating impaired fatty acid oxidation in PT cells lacking TβRII as reflected by respiration (basal and maximal), spare capacity and ATP-linked OCR (n=3 independent biological replicates). (C) p= 0.0012; (d) p= 0.0117 and (e) p= 0.0001. Data are presented as mean values ±SEM. Statistical significance was determined by 2 way ANOVA followed by Sidak's multiple comparisons test with $p<0.05$ considered statistically significant. Dots represent the number of animals per group. * represents $p<0.05$; ** represents $p<0.01$ ***$p<0.001$. Source data are provided as a Source Data file.

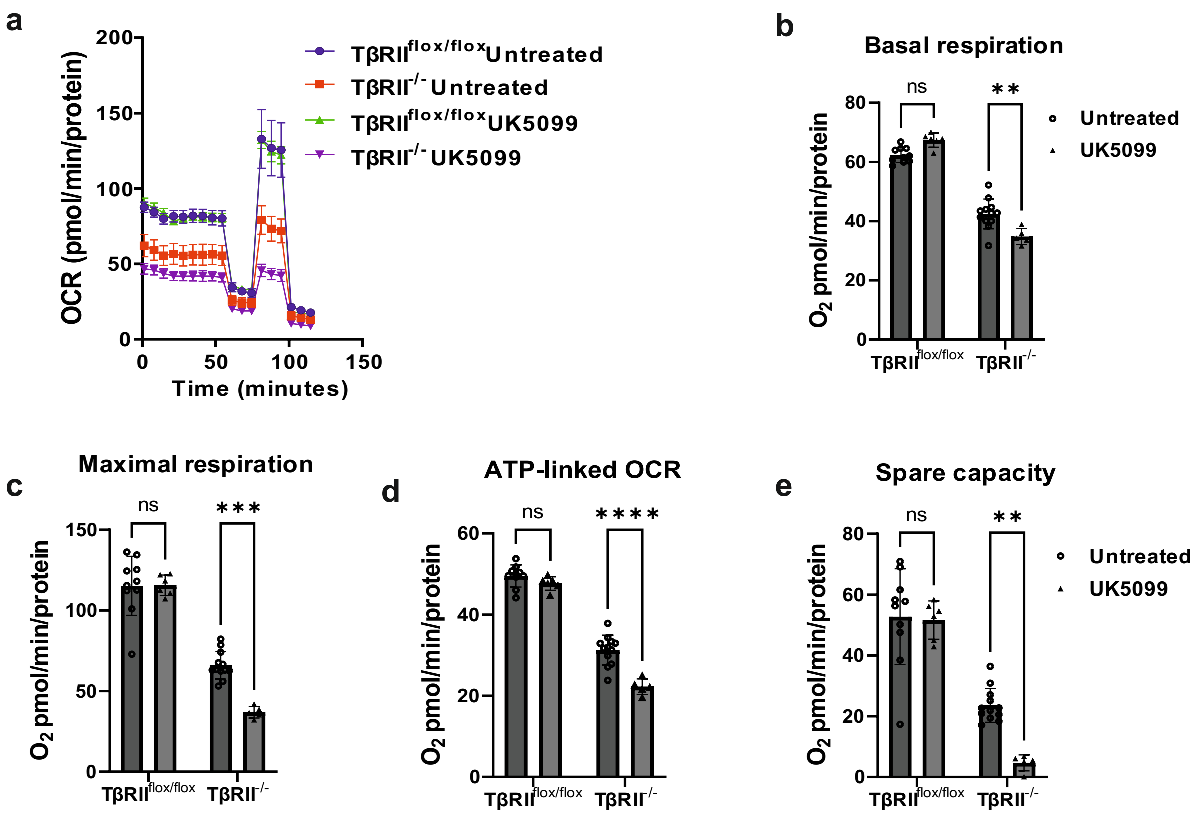

Supplementary Fig. 7: Seahorse analyses of substrate dependency indicating increased glycolytic dependency in TβRII$^{-/-}$ compared to TβRII$^{flox/flox}$ PT cells as reflected by respiration (basal and maximal), spare capacity and ATP-linked OCR (n=3 independent biological replicates). Data are presented as mean values ±SEM. Statistical significance was determined by 2 way ANOVA followed by Sidak's multiple comparisons test with p<0.05 considered statistically significant. (b) Basal respiration (***p*=0.0033). (c) Maximal respiration (****p*=0.0004). (d) ATP-linked OCR (*****p*<0.0001). (e) Spare capacity (***p*=0.0066). * represents p<0.05; ** represents p <0.01. Source data are provided as a Source Data file.

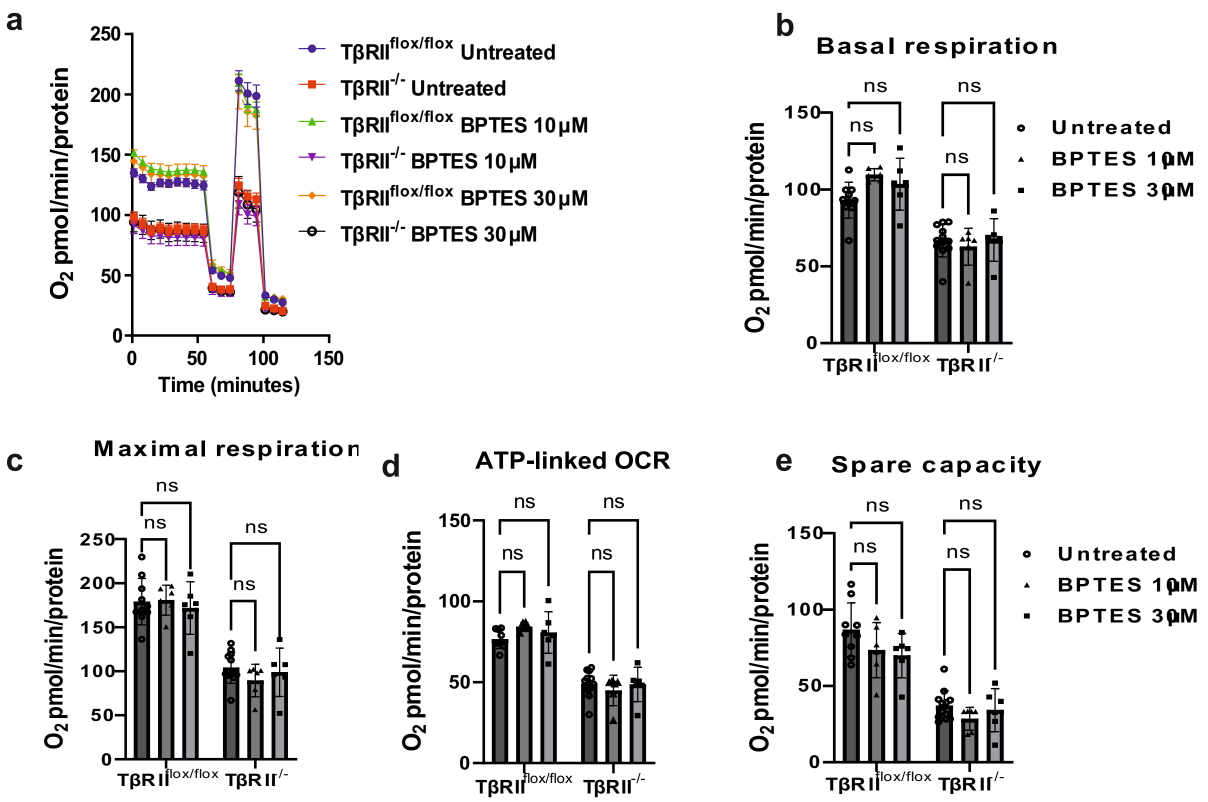

Supplementary Fig. 8: (a-e) Seahorse analysis of substrate dependency indicating absence of glutamine oxidation dependency in PT cells as reflected by respiration (basal and maximal), spare capacity and ATP-linked OCR (n=3 independent biological replicates). Data are presented as mean values ±SEM. Statistical significance was determined by 2 way ANOVA followed by Sidak's multiple comparisons test with $p<0.05$ considered statistically significant. Source data are provided as a Source Data file.

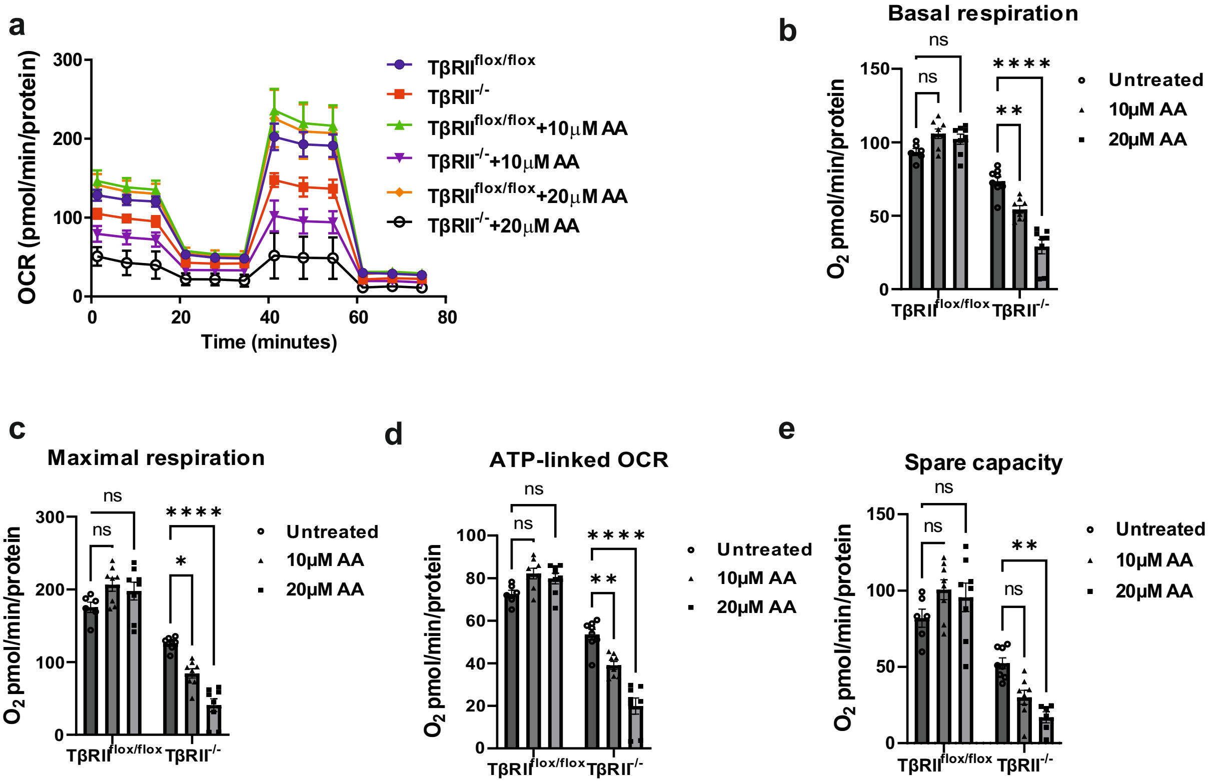

Supplementary Fig. 9: Proximal tubule TβRII deletion increases susceptibility to AA induced mitochondrial dysfunction. (a-e) Treatment of TβRII$^{-/-}$ PT cells with AA (10 or 20 μM) decreased respiration (basal and maximal), spare capacity and ATP-linked OCR compared to TβRII$^{flox/flox}$ PT cells (n=3 independent biological replicates). Data are presented as mean values ±SEM. Statistical significance was determined by 2 way ANOVA followed by Sidak's multiple comparisons test with $p<0.05$ considered statistically significant. (b) Basal respiration (***p*=0.0039 and *****p*<0.0001). (c) Maximal respiration (**p*=0.0141 and *****p*<0.0001). (d) ATP-linked OCR (***p*=0.0041 and *****p*<0.0001). (e) Spare capacity (***p*=0.0052). * represents p<0.05; *** represents p<0.001. Source data are provided as a Source Data file.

a

b

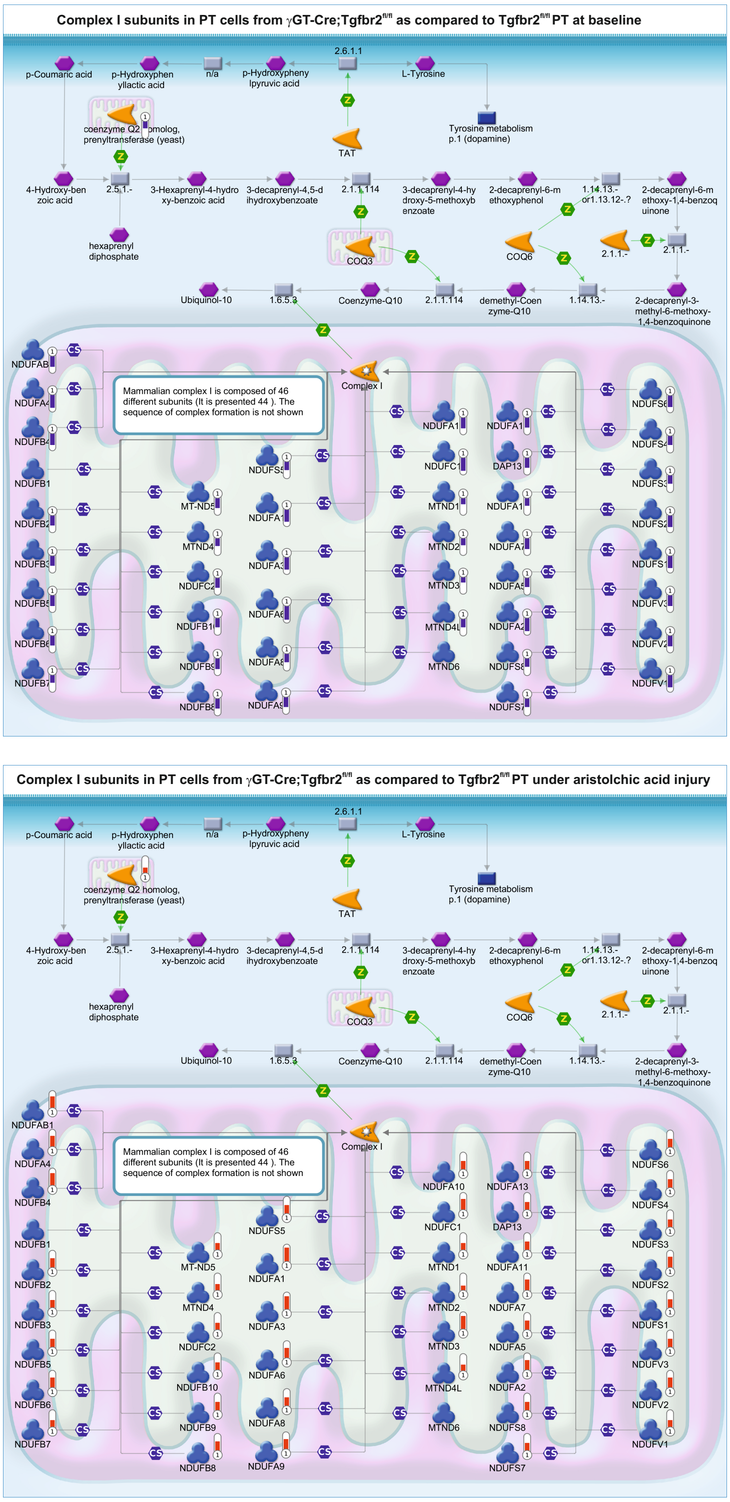

Supplementary Fig. 10: Proximal tubule TβRII deletion impairs ubiquinone metabolism and mitochondrial complex I (a) Metacore overrepresentation pathway analysis of differentially expressed genes in PT clusters showing a decrease of complex I subunits in uninjured γGT-Cre;Tgfbr2$^{fl/fl}$ compared to Tgfbr2$^{fl/fl}$ PT cells. (b) AA injury-induced stress increased the mRNA levels of complex I subunits.

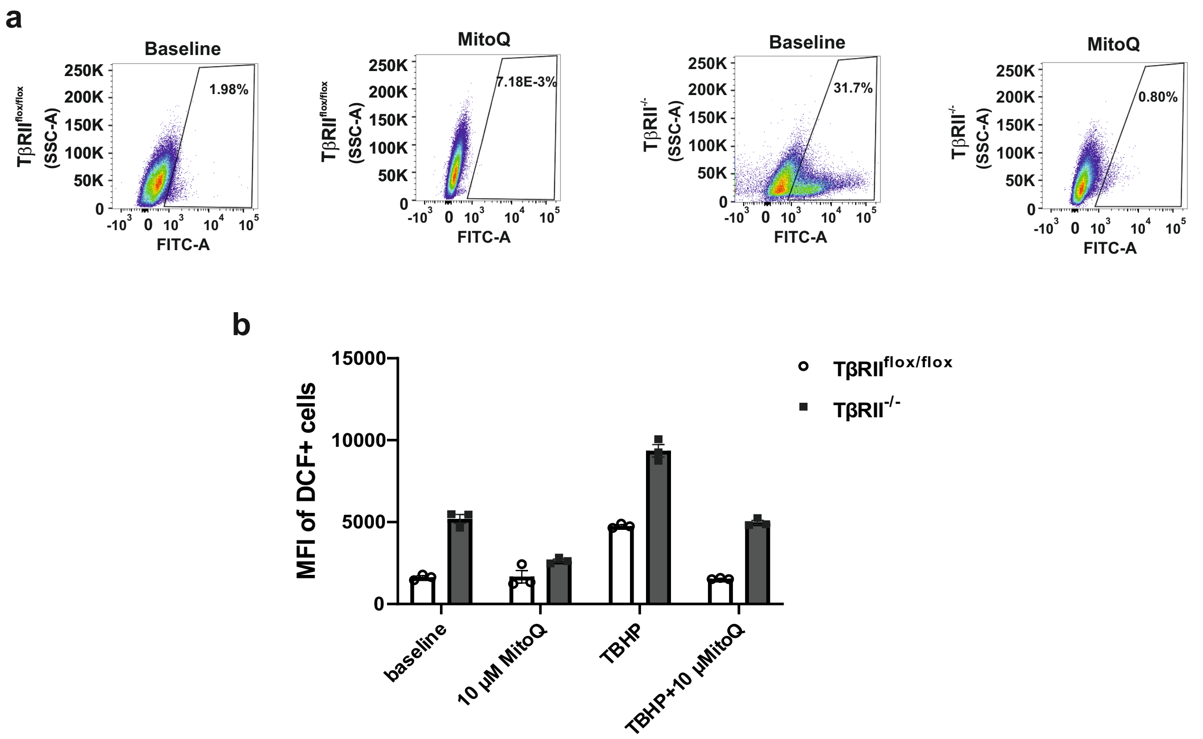

Supplementary Fig. 11: FACS analysis plots and quantification ROS in PT cells treated or not with 10 μM MitoQ. MitoQ treatment decreased ROS in TβRII$^{-/-}$ PT cells to the level of TβRII$^{flox/flox}$ PT cells, but did not affect basal ROS production in TβRII$^{flox/flox}$ PT cells. Tert-Butyl hydroperoxide (TBHP) was used as positive control (3 biological replicates).Data are presented as mean values ±SEM. The dots represent biological replicates. Source data are provided as a Source Data file.

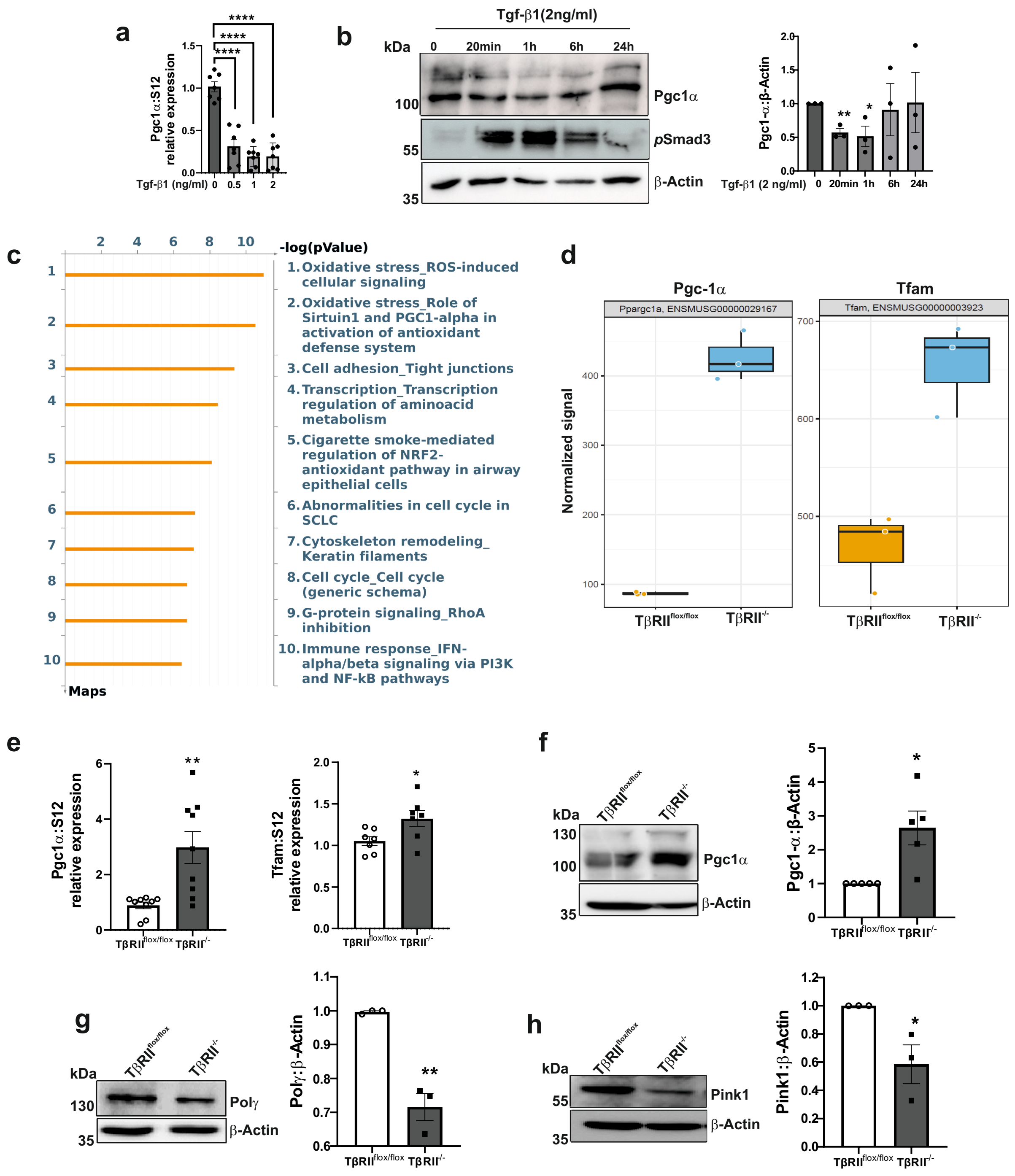

Supplementary Fig. 12: Effect of proximal tubule TGF-β signaling on Pgc1α, Polγ and Pink1. (a) Dose-dependent effect of TGF-β on TβRII$^{flox/flox}$ PT cell Pgc1α mRNA levels measured by RT-qPCR and normalized to S12 mRNA levels (n=7 independent biological replicates, p<0.0001). (b) Immunoblotting and quantification showing the association of transient activation of TGF-β signaling (p-Smad3) with transient repression of Pgc1α protein induced by 2 ng/ml TGF-β1. β-Actin was used as loading and blotting control (n=3 independent experiments, *p= 0.0328 and **p= 0.0018). (c) Overrepresentation analysis using Metacore showing the top 10 significantly

affected in TβRII$^{-/-}$ compared to TβRII$^{flox/flox}$ PT cells. (d) RNAseq normalized Pgc1α and Tfam signals were strikingly increased in TβRII$^{-/-}$ (Pgc1α; minima= 395.6, center= 416.8, maxima= 465.2 and Tfam; minima=601.7, center= 673.1, maxima= 692.1) compared to TβRII$^{flox/flox}$ PT cells (Pgc1α; minima= 85.5, center= 86.1, maxima= 89.3 and Tfam; minima= 421.1, center= 484.4, maxima= 497); n=3 independent biological replicates. (e) Pgc1α and Tfam mRNA levels measured by RT-qPCR and normalized to the mRNA levels of S12 showing basal increase in TβRII$^{-/-}$ compared to TβRII$^{flox/flox}$ PT cells (n=9 independent experiments for Pgc1α, *p*= 0.0026) and (n=7 independent experiments for Tfam, *p*= 0.0308). (f) Immunoblotting and quantification showing basal increase of Pgc1α protein expression in TβRII$^{-/-}$ PT cells as compared to TβRII$^{flox/flox}$ PT cells (n=3 independent experiments, *p*=0.0112). (g) Immunoblotting and quantification showing basal decrease of Polγ protein expression in TβRII$^{-/-}$ compared to TβRII$^{flox/flox}$ PT cells (n=3 independent experiments, *p*=0.0022). (h) Immunoblotting and quantification showing basal decrease of Pink1 protein expression in TβRII$^{-/-}$ compared to TβRII$^{flox/flox}$ PT cells. β-Actin was used as loading and blotting control (n=3 independent experiments, *p*=0.0391). Data are presented as mean values ±SEM. Statistical significance was determined by unpaired Student's *t* test (two groups) with p<0.05 considered statistically significant. * represents *p*<0.05; ** represents *p*<0.01; **** represents *p*<0.0001. Source data are provided as a Source Data file.

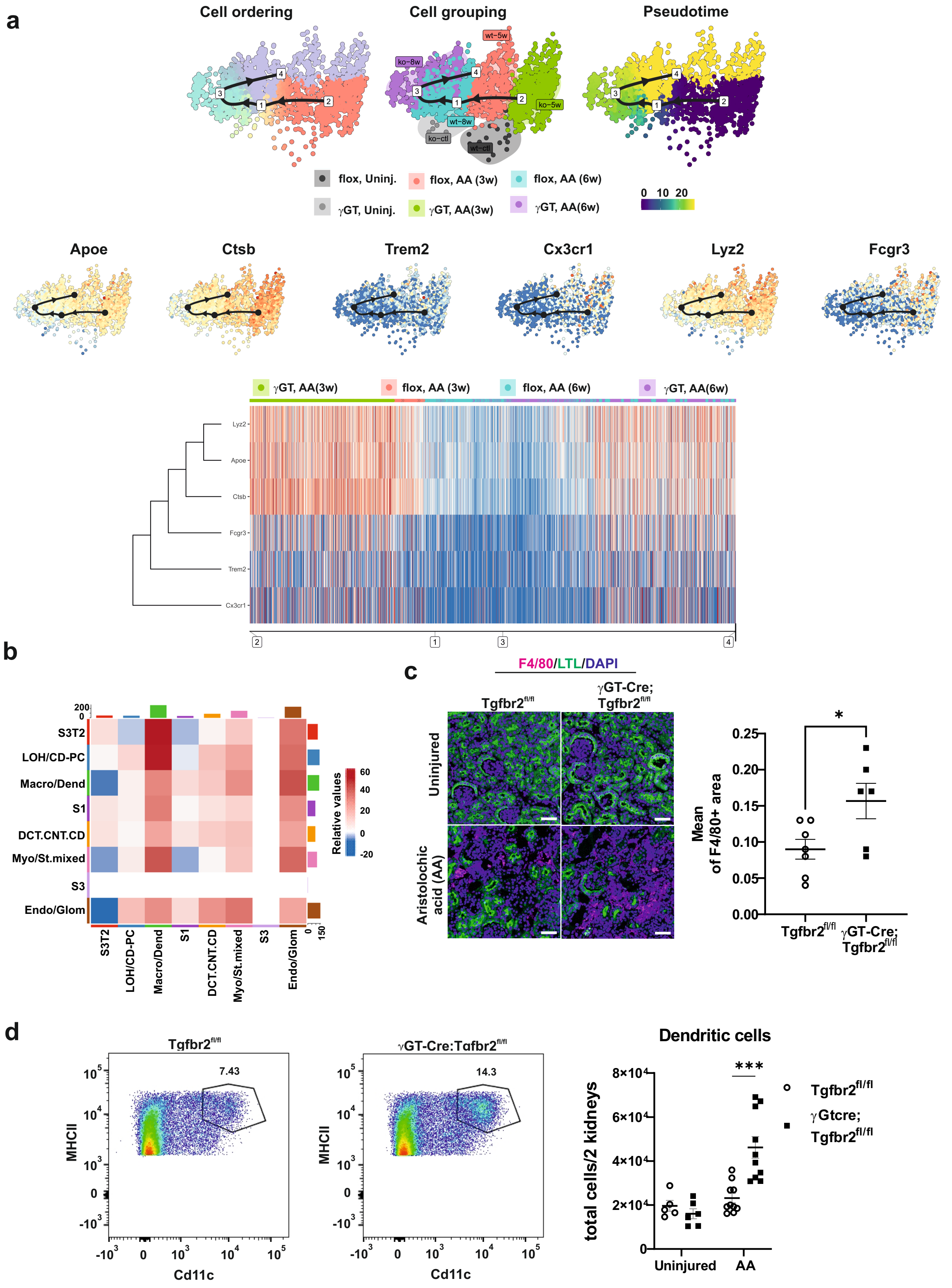

Supplementary Fig. 13: Proximal tubule TβRII deletion increases tubular-macrophage/dendritic cells interaction and infiltrates 3 weeks after AA injury. (a) Trajectory inference performed on *Myo/str. mixed* clusters reveals increased macrophages markers in the

dataset γGT-Cre;Tgfbr2$^{fl/fl}$ 3 weeks after AA injury compared to other conditions (3 weeks Tgfbr2$^{fl/fl}$, 6 weeks γGT-Cre;Tgfbr2$^{fl/fl}$ and 6 weeks Tgfbr2$^{fl/fl}$). The trajectory diagram and heat map show macrophage marker expression at each injury time points. (b) Heat map of cluster interactions, analyzed with Cellchat database of LR pairs, showing increased *S3T2* cells interaction with *Macro/Dend.* cells in γGT-Cre;Tgfbr2$^{fl/fl}$ compared to Tgfbr2$^{fl/fl}$ datasets 3 weeks after AA injury. (c) Representative immunofluorescence images and quantification of F4/80 showing increased macrophage infiltrates in renal cortices of γGT-Cre;Tgfbr2$^{fl/fl}$ mice compared to their Tgfbr2$^{fl/fl}$ littermates 3 weeks after AA injury. Lotus tetragonolobus lectin (LTL) is used as proximal tubule's marker; n=7 (Tgfbr2$^{fl/fl}$) and 6 (γGT-Cre;Tgfbr2$^{fl/fl}$) mice, p=0.0307. (d) FACS analysis of renal interstitial cells showing increased numbers of dendritic cells in the kidneys of γGT-Cre;Tgfbr2$^{fl/fl}$ mice compared to those from their Tgfbr2$^{fl/fl}$ littermates 3 weeks after AA injury (n=5 uninjured, 10 injured (Tgfbr2$^{fl/fl}$) and 6 uninjured, 10 injured γGT-Cre;Tgfbr2$^{fl/fl}$ mice, injured p=0.0002). Data are presented as mean values ±SEM. Statistical significance was determined by unpaired Student's *t* test (two groups) or 2 way ANOVA followed by Sidak's multiple comparisons test with $p<0.05$ considered statistically significant. Dots represent the number of animals per group. * represents $p<0.05$; ***$p<0.001$. Source data are provided as a Source Data file.

a

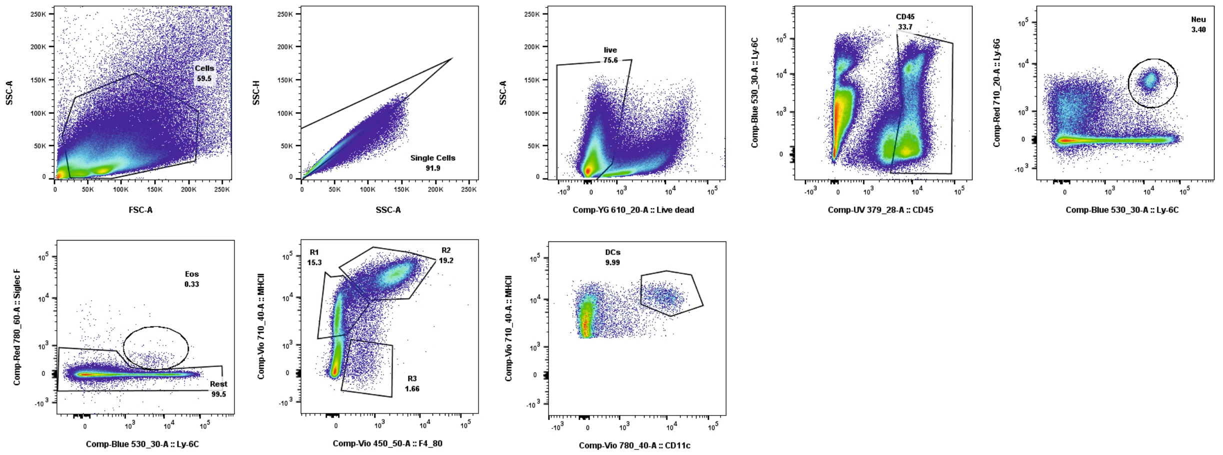

b

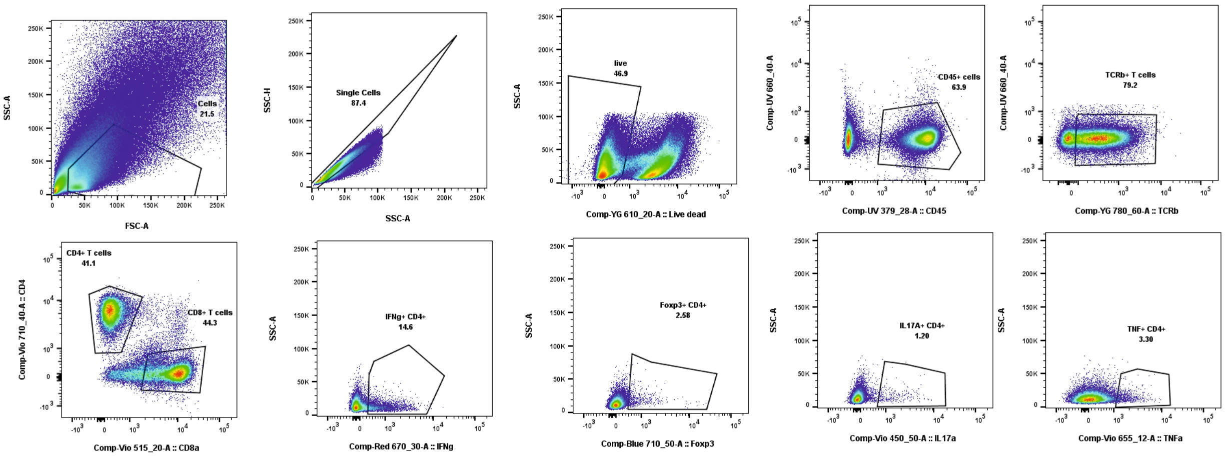

Supplementary Fig. 14: Myeloid and lymphoid cell FACS gating strategies. (a). Myeloid cells gating strategy depicting identification of dendritic cell and other cell types. (b) Lymphoid cells gating strategy showing identification of CD4+, CD8+ and their subpopulations.

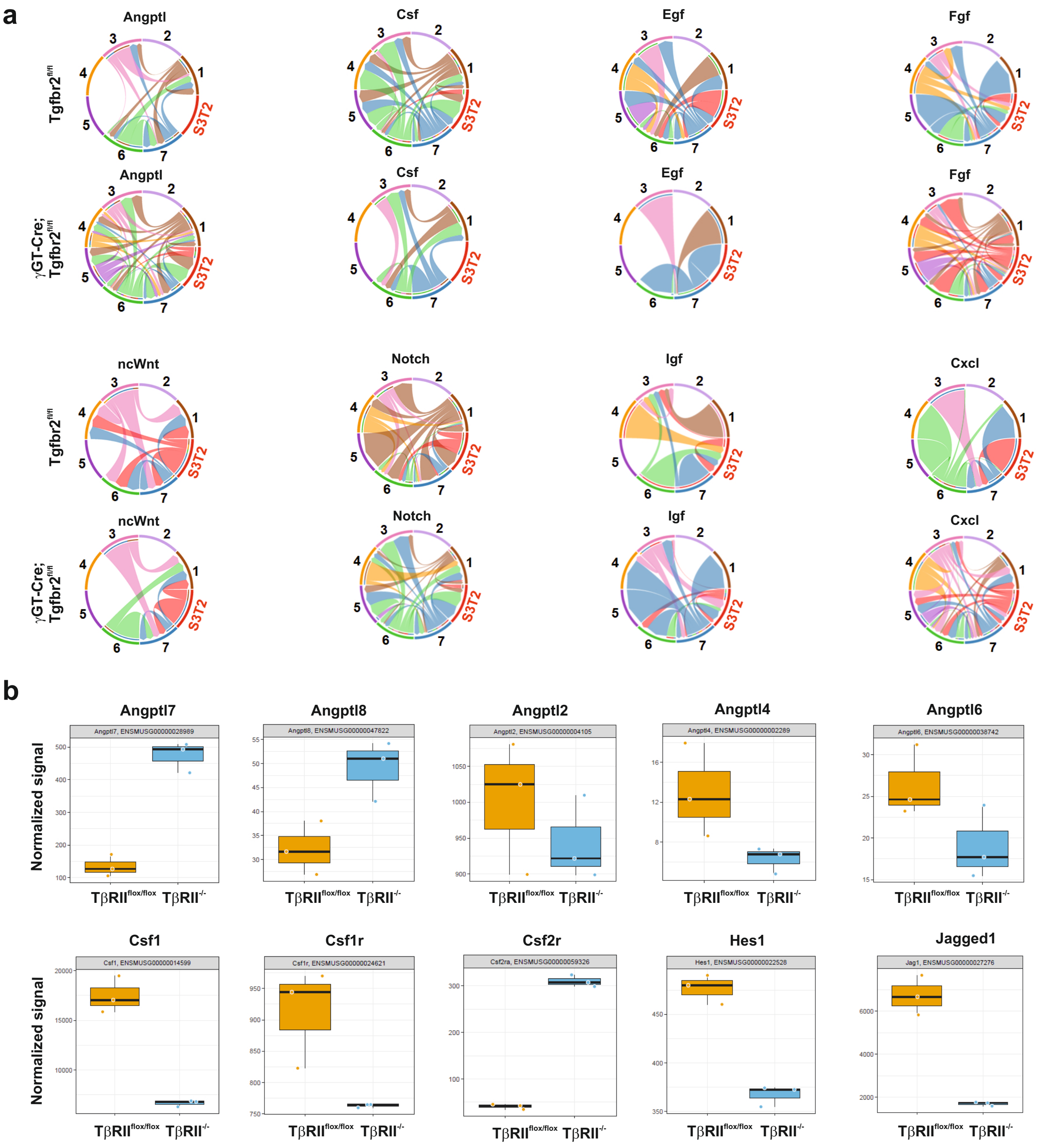

Supplementary Fig. 15: Cellchat analyses of differentially expressed genes involved in *S3T2-Macro/dend.* interactions. (a) Diagram showing factors mediating outgoing and incoming interactions between clusters. (b) RNAseq normalized signals of selected adaptive and maladaptive factors (Minima, center and maxima values are in Source Data file; n= 3 independent biological replicates). Numbers are 1: endothelial and glomerular cells (Endo/Glom); 2: S3 PT cells; 3: myofibroblasts and stromal cells (Myo/St. mixed); 4: distal convoluted tubular, connecting and intercalated cells (DCT.CNT.CD-IC); 5: S1 PT cells; 6: macrophages and dendritic cells (Macro/Dend.); 7: loop of Henle and principal cells (LOH/CD-

PC). Source data are provided as a Source Data file.

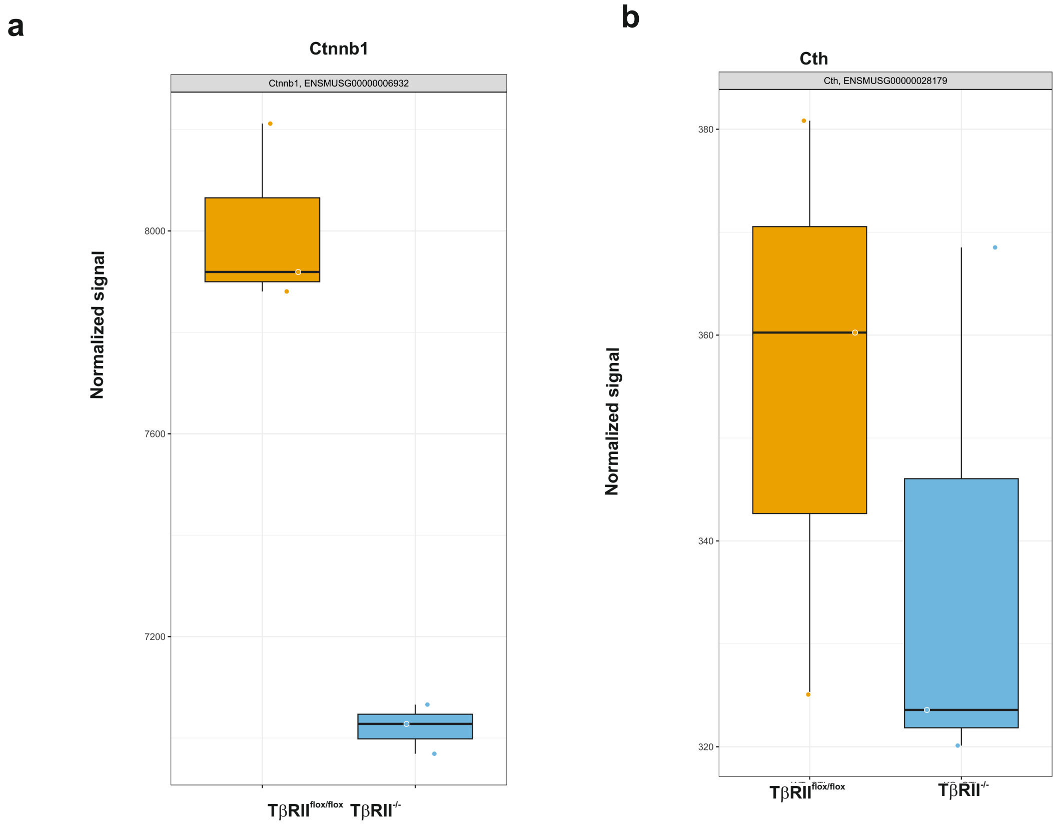

Supplementary Fig. 16: (a, b) Potential adaptive factors, β-catenin (Ctnnb1) and Cystathionase (Cth) are decreased in TβRII$^{-/-}$ (Ctnnb1; minima= 6969, center= 7027.9, maxima= 7066 and Cth; minima= 320.1, center= 323.6, maxima= 368.5) compared to TβRII$^{flox/flox}$ PT cells (Ctnnb1; minima= 7880.6, center= 7919, maxima= 8211.5 and Cth; minima= 325.1, center= 360.2, maxima= 380.8) as assessed by RNAseq (n=3 independent biological replicates). Source data are provided as a Source Data file.

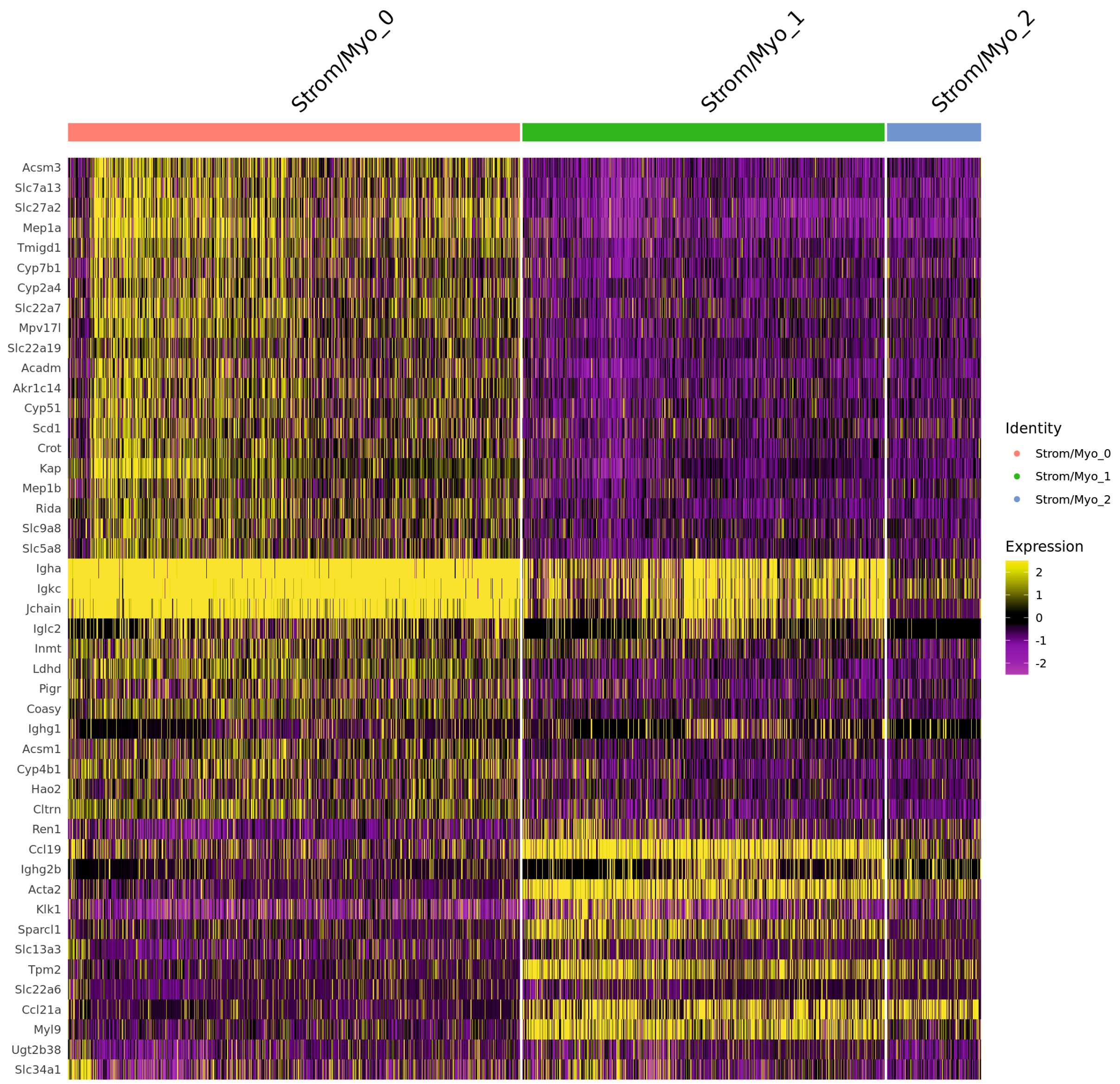

Supplementary Fig. 17: Sub-clustering analysis of *Myo/Str. mixed* identified 3 subpopulations. Heatmap showing the 3 sub-populations (Strom/Myo_0, Strom/Myo_1 and Strom/Myo_2) and significantly up and down-regulated markers per subgroups.

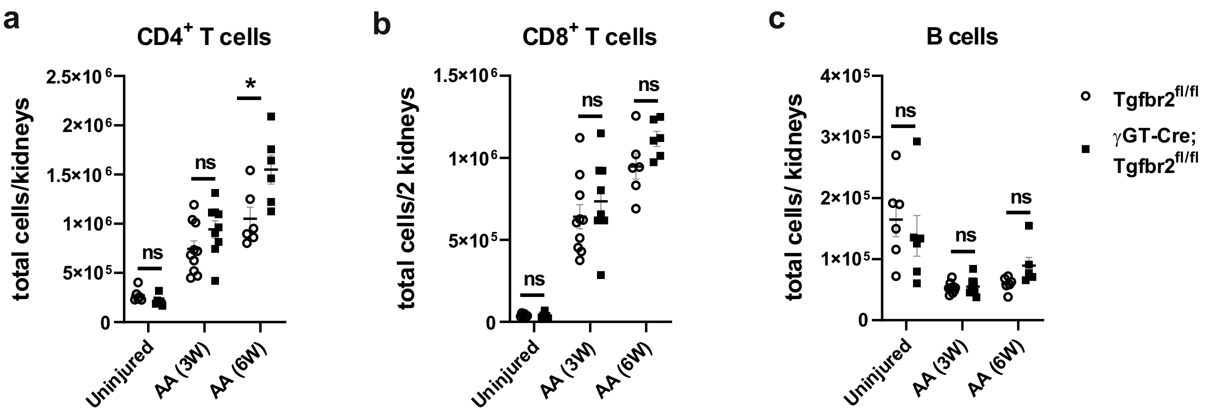

Supplementary Fig. 18: (a-c) FACS analyses of renal leukocytes showing no significant difference of leukocytes 3 weeks after AA injury and significant increase of CD4+ T cell number in kidneys of γGT-Cre;Tgfbr2$^{fl/fl}$ mice compared to those from their Tgfbr2$^{fl/fl}$ littermates 6 weeks after AA injury. CD8+ T cell numbers were increased in Tgfbr2$^{fl/fl}$ compared to γGT-Cre;Tgfbr2$^{fl/fl}$ kidneys, but did not reach statistical significance 6 weeks after AA injury. Uninjured (n= 6 Tgfbr2$^{fl/fl}$ and 6 γGT-Cre;Tgfbr2$^{fl/fl}$), 3 weeks (n= 9 Tgfbr2$^{fl/fl}$ and 8 γGT-Cre;Tgfbr2$^{fl/fl}$) and 6 weeks (n=6 Tgfbr2$^{fl/fl}$ and 6 γGT-Cre;Tgfbr2$^{fl/fl}$, CD4+ T cells *p*=0.0205) mice. Data are presented as mean values ±SEM. Statistical significance was determined by 2 way ANOVA followed by Sidak's multiple comparisons test with p<0.05 considered statistically significant. The dots represent the number of animals per group. *p represents <0.05. Source data are provided as a Source Data file.

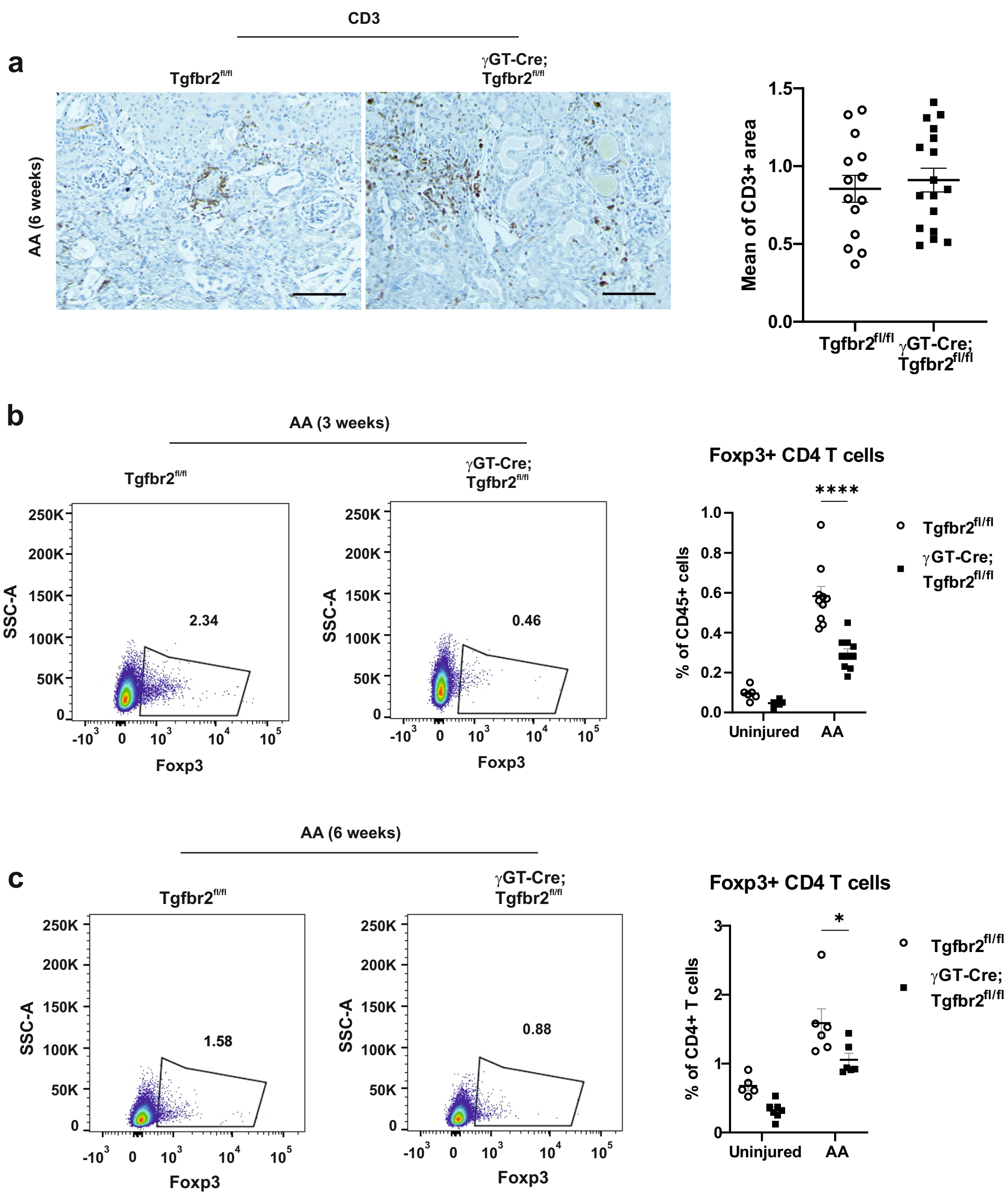

Supplementary Fig. 19: Proximal tubule TβRII deletion decreases the number of Foxp3+ CD4 T cells after AA induced injury. (a) Representative image and quantification showing no difference in CD3+ cell infiltrate in renal cortices of γGT-Cre;Tgfbr2$^{fl/fl}$ and floxed littermate mice; n=14 (Tgfbr2$^{fl/fl}$) and 17 (γGT-Cre;Tgfbr2$^{fl/fl}$) mice. Scale bars represent 200 μm. (b, c) FACS analyses of the Foxp3+ CD4 T cell percentage of the CD45+ cells 3 weeks (n=6 uninjured, 10 injured Tgfbr2$^{fl/fl}$ and 6 uninjured, 10 injured γGT-Cre;Tgfbr2$^{fl/fl}$ mice, injured *p*<0.0001) (b) and 6 weeks (n=5 uninjured, 6 injured Tgfbr2$^{fl/fl}$ and 7 uninjured, 6 injured γGT-Cre;Tgfbr2$^{fl/fl}$ mice, injured *p*=0.0328) (c) after AA injury. Data are presented as mean values ±SEM. Statistical significance was determined by unpaired Student's *t* test (two groups) or 2 way ANOVA followed

by Sidak's multiple comparisons test with p<0.05 considered statistically significant. The dots represent the number of animals per group. * represents p<0.05; **** represents p<0.0001. Source data are provided as a Source Data file.

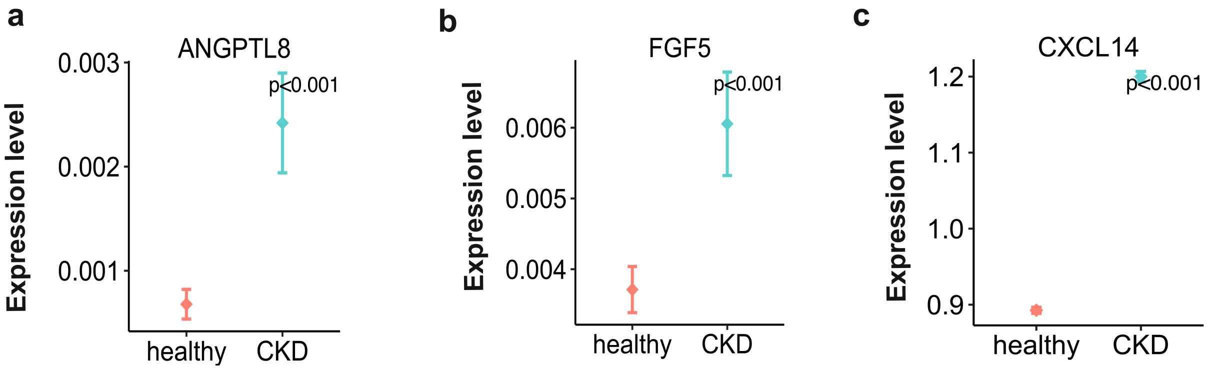

Supplementary Fig. 20: (a-c) Differential gene expression analysis showing a significant increase of potential maladaptive factors (ANGPTL8, FGF5 and CXCL14) in CKD PT cells as compared to healthy PT cells from the snRNAseq online database. N= 5 CKD patients (eGFR<60) and 3 healthy controls. Differential gene expression was evaluated using the Wilcoxon Rank Sum test from the *FindMarkers* function. (a) p= 0.000003; (b) p= 0.00093617 and (c) p=0. Source data are provided as a Source Data file.

Used Reagents and Antibodies

	Company	Cat. number	Dilution
**AA Injury Models**			
Aristolochic acid	Sigma	A9451	
**Staining**			
10% Formalin	Sigma	HT501128	
Oil red O	Sigma	O0625	
Sirius red	Waldeck, Münster, Germany		
H&E Staining Kit (Hematoxylin and Eosin)	Abcam	ab245880	
VECTASTAIN Elite ABC Kit	Vector Laboratories	PK-6200	
H2O2	Sigma	H1009	
DAB	Vector Laboratories	SK-4105-L120	
Citrate Buffer	Sigma	C9999	
Normal goat serum	Jackson Immuno research	005-000-121	
Normal donkey serum	Jackson Immuno research	017-000-122	
LowCross-Buffer	Candor	100 050	
Antifade Monting medium with Dapi	Vector Laboratories	H-1800	
**Spatial transcriptomics**			
Visium Spatial Gene Expression	10X Genomics		
DNA 12000 Kit	Agilent	5067-1508	
High Sensitivity D5000 ScreenTape	Agilent	5067-5592	
High Sensitivity D5000 Reagents	Agilent	5067-5593	
SPRIselect Reagent	Beckman Coulter	4700273718	
**Flow cytometry**			
Collagenase IV	Worthington	LS004188	
Percoll	Sigma-Aldrich	GE17-0891	
**Mitochondria fractionation**			
Cell mitochondria isolation kit	ThermoFisher	89874	
Protease inhibitor coktail	Roche	11697498001	
**Cell culture**			
DMEM/F12	Corning	10-092-CV	
FBS	Gibco	10270-106	
**PT supplements**			
Hydrocortisone	Sigma Aldrich	H0135	
ITS (insulin/transferrin/selenium)	Sigma Aldrich	I1884-1	
T3	Sigma Aldrich	T5516	
Interferon-gamma	Sigma Aldrich	I4777	
TGF-beta 1 Protein	R&D Systems	101-B1-001/CF<br>7754-BH-005/CF	

**Seahorse**		
Palmitate Oxidation Stress Test Kit	Bucher	103693-100
Mito Stress Test Kit	Bucher	103015-100
Glucose/Pyruvate Oxidation Stress Test Kit	Bucher	103673-100
Glutamine Oxidation Stress Test Kit	Bucher	103674-100
L-carnitine	Agilent	6496363
Etomoxir	Sigma	E1905
Oligomycin	Sigma	75351
FCCP	Sigma	C2920
Rotenone/Antimycin	Sigma	R8875
BPTES	Sigma	SML0601
UK5099	Sigma	PZ0160
Passive lysis buffer	Promega	E1941
Bradford Assay reagents	Biorad	5000002
Pierce BCA Protein Assay A	Thermo Scientific	23228
Pierce BCA Protein Assay B	Thermo Scientific	1859078
**ATP, Lactate and NAD+/NADH assay**		
ATP assay	Promega	G7570
Lactate essay	Promega	J5021
NAD+/NADH essay	Promega	G9071
**DCFDA assay**		
MitoQ	Focus Biomolecules	10-1363-25
NAD+	Sigma-Aldrich	N8285<br>N1636
**qPCR**		
Nucleospin RNA extraction kit	Macherey Nagel	740955.25
AffinityScript Multi-Temp RT kit	Agilent	600107
KAPA SYBR FAST	Roche	KK4618
**Immunoblotting**		
EDTA	Sigma	27285
NP-40 Substitute	Sigma	74385
Na3VO4	Sigma	S6508
Protease inhibitor cocktails	Roche	11 836 170 001
PMSF	Sigma	P 7626
Aprotinin	Sigma	A1153
Leupeptin	Sigma	L2884
Pepstatin	Sigma	P 4265
BSA	Carl Roth	8076.4
HRP substrate	PerkinElmer	NEL113001EA
**Antibodies**		
**IF/IHC**		

Pgc1a (Monoclonal, 4C1.3)	Merck	ST1202	1:500
CD3 (Monoclonal, SP7)	Abcam	ab16669	1:150
F4/80 (Monoclonal, CI:A3-1)	Abcam	ab6640	1:150
Kim-1 (Polyclonal)	R&D Systems	AF1817	1:200
LTL	Vector Laboratories	FL-1321	1:400
Secondary AB, Alexa fluor	Life technology		
**Immunoblotting**			
Pink1 (Polyclonal)	Novus	BC100-494	1:1000
LC3A (Monoclonal, D50G8)	Cell Signaling	4599S	1:500
Pgc1a (Monoclonal, 4C1.3)	Merck	ST1202	1:500
Phospho-Smad3 (Polyclonal)	Rockland	600-401-919	1:500
Polg (Monoclonal, G-6)	Santa Cruz	sc-390634	1:200
Tom20 (Polyclonal)	Santa Cruz	sc-11415	1:1000
alpha-tubulin (Monoclonal, GT114)	GenTex	GTX628802-01	1:4000
Beta-actin (Monoclonal, AC-15)	Sigma	A5441	1:20000
Total OXPHOS Antibody Cocktail (5 mAbs)	Abcam	ab110413	1:1000
Anti-NDUFB8 antibody [20E9DH10C12] (ab110242)			
Anti-SDHB antibody [21A11AE7] (ab14714)			
Anti-UQCRC2 antibody [13G12AF12BB11] (ab14745)			
Anti-MTCO1 antibody [1D6E1A8] (ab14705)			
Anti-ATP5A antibody [15H4C4] (ab14748)			
polyclonal goat anti-rabbit-HRP	Pierce	31430	
polyclonal goat anti-mouse-HRP	Pierce	31460	

List of antibodies used in FACS					
**Fluorochrome**	**Target**	**Clone**	**Reseller**	**Cat #**	**Dilution (1/x)**
BUV395	CD45	30-F11	BD Horizon	564279	300
BV785	CD11c	N418	BioLegend	117336	300
BV711	MHC II	M5/114.15.2	BioLegend	107643	1000
BV650	XCR1	ZET	BioLegend	148220	200
BV605	SA	strepdavidin	BioLegend	405229	300
BV510	CD11b	M1/70	BioLegend	101263	100
PerCP-Cy5.5	MGL2	URA1	BioLegend	146810	300
FITC	Ly-6C	HK1.4	BioLegend	128006	300
PE-Cy7	CD103	2E7	BioLegend	121426	200
PE	PDL2	TY25	BioLegend	107205	300
APC-Cy7	SiglecF	E50-2440	BD Pharmingen	565527	300
AF700	Ly-6G	1A8	BioLegend	127622	400
APC	CD64	X54-5/7.1	BioLegend	139306	100
Biotin	SIRPa	P84	BioLegend	144026	100
BUV661	CD3	145-2C11	BD OptiBuild	750638	400
BV785	CD90.2	30-H12	BioLegend	105331	1000
BV711	CD4	RM4-5	BioLegend	100550	100
BV650	CD19	6D5	BioLegend	115541	300
BV605	CD25	PC61	BioLegend	102036	200
BV421	CD11b	M1/70	BioLegend	101251	300
BV421	CD11c	N418	BioLegend	117343	300
BV421	F4/80	BM8	BioLegend	123137	300
BV421	GR-1	RB6-8C5	BioLegend	108445	300
BV421	TER-119	TER-119	BioLegend	116234	300
PerCP-eF710	KLRG1	2F1	Invitrogen	46-5893-82	300
FITC	Ki67	11F6	Biolegend	151212	300
PE-Cy7	NKp46	29A1.4	BioLegend	137618	200
PE	ST2	DIH4	BioLegend	146607	300
APC-F750	TCR gd	GL3	BioLegend	118136	150
AF700	TCR b	H57-597	BioLegend	109224	400
APC	DX5	DX5	BioLegend	108910	100
**Viability dye**					
Zombie Red	dead cells		BioLegend	423110	1500

Report of sequencing QC stats

	Name	Number of Spots Under Tissue
o25586_1_1-wt-ctl	wt-ctl	3146
o25586_1_2-ko-ctl	ko-ctl	3411
o25586_1_3-wt-5w	wt-5w	3515
o25586_1_4-ko-5w	ko-5w	3872
o25586_1_6-ko3-8w	ko-8w	3347
o25586_1_7-wt5-8w	wt-8w	3320

	Name	Number of Reads	Mean Reads per Spot
o25586_1_1-wt-ctl	wt-ctl	256055078	81390.67959
o25586_1_2-ko-ctl	ko-ctl	193407956	56701.24773
o25586_1_3-wt-5w	wt-5w	214021389	60888.01963
o25586_1_4-ko-5w	ko-5w	291406727	75260.00181
o25586_1_6-ko3-8w	ko-8w	224717809	67140.06842
o25586_1_7-wt5-8w	wt-8w	231795128	69817.80964

	Name	Mean Reads Under Tissue per Spot	Fraction of Spots Under Tissue
o25586_1_1-wt-ctl	wt-ctl	51496.6691	0.630208333
o25586_1_2-ko-ctl	ko-ctl	36446.04134	0.683293269
o25586_1_3-wt-5w	wt-5w	50968.03812	0.704126603
o25586_1_4-ko-5w	ko-5w	60087.7079	0.775641026
o25586_1_6-ko3-8w	ko-8w	46411.46011	0.670472756
o25586_1_7-wt5-8w	wt-8w	51555.46024	0.665064103

	Name	Median Genes per Spot	Median UMI Counts per Spot
o25586_1_1-wt-ctl	wt-ctl	4234.5	16670
o25586_1_2-ko-ctl	ko-ctl	3244	10801
o25586_1_3-wt-5w	wt-5w	4321	14337
o25586_1_4-ko-5w	ko-5w	5178	19197
o25586_1_6-ko3-8w	ko-8w	3655	9007
o25586_1_7-wt5-8w	wt-8w	3299.5	8285

	Name	Median Genes per Spot	Median UMI Counts per Spot
o25586_1_1-wt-ctl	wt-ctl	4234.5	16670
o25586_1_2-ko-ctl	ko-ctl	3244	10801
o25586_1_3-wt-5w	wt-5w	4321	14337
o25586_1_4-ko-5w	ko-5w	5178	19197
o25586_1_6-ko3-8w	ko-8w	3655	9007
o25586_1_7-wt5-8w	wt-8w	3299.5	8285

	Name	Median Genes per Spot	Median UMI Counts per Spot
o25586_1_1-wt-ctl	wt-ctl	4234.5	16670
o25586_1_2-ko-ctl	ko-ctl	3244	10801
o25586_1_3-wt-5w	wt-5w	4321	14337
o25586_1_4-ko-5w	ko-5w	5178	19197

o25586_1_6-ko3-8w	ko-8w	3655	9007
o25586_1_7-wt5-8w	wt-8w	3299.5	8285

	Name	Valid Barcodes	Valid UMIs
o25586_1_1-wt-ctl	wt-ctl	0.96230914	0.99842514
o25586_1_2-ko-ctl	ko-ctl	0.963811225	0.998520304
o25586_1_3-wt-5w	wt-5w	0.963844684	0.998216459
o25586_1_4-ko-5w	ko-5w	0.963812864	0.999101421
o25586_1_6-ko3-8w	ko-8w	0.960277741	0.999395193
o25586_1_7-wt5-8w	wt-8w	0.957380895	0.999561354

	Name	Sequencing Saturation	Q30 Bases in Barcode
o25586_1_1-wt-ctl	wt-ctl	0.580787016	0.945740689
o25586_1_2-ko-ctl	ko-ctl	0.587929653	0.947285749
o25586_1_3-wt-5w	wt-5w	0.634684432	0.948436636
o25586_1_4-ko-5w	ko-5w	0.618744854	0.949343766
o25586_1_6-ko3-8w	ko-8w	0.740219229	0.949567813
o25586_1_7-wt5-8w	wt-8w	0.801069095	0.950086734

	Name	Sequencing Saturation	Q30 Bases in Barcode
o25586_1_1-wt-ctl	wt-ctl	0.580787016	0.945740689
o25586_1_2-ko-ctl	ko-ctl	0.587929653	0.947285749
o25586_1_3-wt-5w	wt-5w	0.634684432	0.948436636
o25586_1_4-ko-5w	ko-5w	0.618744854	0.949343766
o25586_1_6-ko3-8w	ko-8w	0.740219229	0.949567813
o25586_1_7-wt5-8w	wt-8w	0.801069095	0.950086734

	Name	Q30 Bases in RNA Read	Q30 Bases in UMI
o25586_1_1-wt-ctl	wt-ctl	0.924531035	0.942876603
o25586_1_2-ko-ctl	ko-ctl	0.924562883	0.94432795
o25586_1_3-wt-5w	wt-5w	0.923123048	0.945344344
o25586_1_4-ko-5w	ko-5w	0.928655982	0.946804633
o25586_1_6-ko3-8w	ko-8w	0.932454876	0.947422186
o25586_1_7-wt5-8w	wt-8w	0.932366753	0.947881975

RM= Reads Mapped

	Name	RM to Genome	RM Confidently to Genome
o25586_1_1-wt-ctl	wt-ctl	0.869175955	0.842248495
o25586_1_2-ko-ctl	ko-ctl	0.865237064	0.83669041
o25586_1_3-wt-5w	wt-5w	0.85405112	0.824711127
o25586_1_4-ko-5w	ko-5w	0.875439451	0.844984557
o25586_1_6-ko3-8w	ko-8w	0.905486614	0.877937445
o25586_1_7-wt5-8w	wt-8w	0.947664133	0.922604111

	Name	RM Confidently to Intergenic Regic	RM Confidently to Intronic Regions
o25586_1_1-wt-ctl	wt-ctl	0.019555988	0.013258667

o25586_1_2-ko-ctl	ko-ctl	0.020876085	0.014521254
o25586_1_3-wt-5w	wt-5w	0.0195584	0.015620135
o25586_1_4-ko-5w	ko-5w	0.020353013	0.015381831
o25586_1_6-ko3-8w	ko-8w	0.021253077	0.012090604
o25586_1_7-wt5-8w	wt-8w	0.022182662	0.012365346

	**Name**	**RM Confidently to Exonic Regions**	**RM Confidently to Transcriptome**
o25586_1_1-wt-ctl	wt-ctl	0.80943384	0.788727588
o25586_1_2-ko-ctl	ko-ctl	0.801293071	0.780388972
o25586_1_3-wt-5w	wt-5w	0.789532592	0.769288643
o25586_1_4-ko-5w	ko-5w	0.809249712	0.78880806
o25586_1_6-ko3-8w	ko-8w	0.844593763	0.822598239
o25586_1_7-wt5-8w	wt-8w	0.888056103	0.863456608

	**Name**	**RM Antisense to Gene**	**Fraction Reads in Spots Under Tissue**
o25586_1_1-wt-ctl	wt-ctl	0.006559706	0.656598495
o25586_1_2-ko-ctl	ko-ctl	0.007157177	0.654459588
o25586_1_3-wt-5w	wt-5w	0.007004842	0.870071277
o25586_1_4-ko-5w	ko-5w	0.006450812	0.825810692
o25586_1_6-ko3-8w	ko-8w	0.006733752	0.722138807
o25586_1_7-wt5-8w	wt-8w	0.009899992	0.773334206

	**Name**	**Total Genes Detected**
o25586_1_1-wt-ctl	wt-ctl	18293
o25586_1_2-ko-ctl	ko-ctl	17715
o25586_1_3-wt-5w	wt-5w	18690
o25586_1_4-ko-5w	ko-5w	19061
o25586_1_6-ko3-8w	ko-8w	17909
o25586_1_7-wt5-8w	wt-8w	17598

After QC analysis and additional filtering

o25586_1_1-wt-ctl-space	wt-ctl	2187
o25586_1_2-ko-ctl-space	ko-ctl	2423
o25586_1_3-wt-5w-spac	wt-5w	2770
o25586_1_4-ko-5w-spac	ko-5w	2934
o25586_1_6-ko3-8w-spa	ko-8w	2261
o25586_1_7-wt5-8w-spa	wt-8w	2549

Total number of spots: 15124
Median UMIs: 14774
Median genes: 4374
Median mitochondrial percentage: 13.050

MetaCore Analysis Report of Complex I in S3T2 PT

MetaCore Data					S3T2 Ko vs WT ctl_avg_log2FC	
#	Input IDs	Network Object Name	Gene Symbol	Description	Signal	p-value
1		2-Decaprenyl-6-methoxy-1,4-benzoquinone intracellular anatomical structure		2-Decaprenyl-6-methoxy-1,4-benzoquinone		
2		2-Decaprenyl-6-methoxyphenol intracellular anatomical structure		2-Decaprenyl-6-methoxyphenol		
3		2-decaprenyl-3-methyl-6-methoxy-1,4-benzoquinone intracellular anatomical structure		2-decaprenyl-3-methyl-6-methoxy-1,4-benzoquinone		
4		2.1.1.-				
5		3-Decaprenyl-4,5-dihydroxybenzoate intracellular anatomical structure		3-Decaprenyl-4,5-dihydroxybenzoate		
6		3-Decaprenyl-4-hydroxy-5-methoxybenzoate intracellular anatomical structure		3-Decaprenyl-4-hydroxy-5-methoxybenzoate		

7		3-Hexaprenyl-4-hydroxy-benzoic acid intracellular anatomical structure		3-Hexaprenyl-4-hydroxy-benzoic acid		
8		4-Hydroxy-benzoic acid intracellular anatomical structure		4-Hydroxy-benzoic acid		
9		COQ3	Coq3	Ubiquinone biosynthesis O-methyltransferase, mitochondrial		
10		COQ6	Coq6	Ubiquinone biosynthesis monooxygenase COQ6, mitochondrial		
11		Coenzyme-Q10 intracellular anatomical structure		Coenzyme-Q10		
12	**Ndufa12**	DAP13	Ndufa12	NADH dehydrogenase [ubiquinone] 1 alpha subcomplex	-0.490431	0
13		H('+) + Coenzyme-Q10 + NADH = Ubiquinol-10 + NAD('+)				
14		H('+) + NAD(P)H + O(,2) + 2-decaprenyl-3-methyl-6-methoxy-1,4-benzoquinone = NADP('+) + demethyl-Coenzyme-Q10 + H(,2)O				

15		H('+) + NAD(P)H + p-Hydroxyphenylpyruvic acid = NADP('+) + p-Hydroxyphenyllactic acid				
16		L-Aspartic acid + p-Hydroxyphenylpyruvic acid = L-Tyrosine + Oxaloacetic acid				
17		L-Tyrosine intracellular anatomical structure		L-Tyrosine		
18	**mt-Nd5**	MT-ND5	ND5	NADH-ubiquinone oxidoreductase chain 5	-0.500391	0
19	**mt-Nd1**	MTND1	ND1	NADH-ubiquinone oxidoreductase chain 1	-0.312574	0
20	**mt-Nd2**	MTND2	ND2	NADH-ubiquinone oxidoreductase chain 2	-0.345866	0
21	**mt-Nd3**	MTND3	ND3	NADH-ubiquinone oxidoreductase chain 3	-0.253271	0
22	**mt-Nd4**	MTND4	ND4	NADH-ubiquinone oxidoreductase chain 4	-0.37974	0
23	**mt-Nd4l**	MTND4L	ND4L	NADH-ubiquinone oxidoreductase chain 4L	-0.431259	0

24		MTND6	ND6	NADH-ubiquinone oxidoreductase chain 6		
25	**Ndufa1**	NDUFA1	Ndufa1	NADH dehydrogenase [ubiquinone] 1 alpha subcomplex subunit 1	-0.638382	0
26	**Ndufa10**	NDUFA10	Ndufa10	NADH dehydrogenase [ubiquinone] 1 alpha subcomplex subunit 10,	-0.526248	0
27	**Ndufa11**	NDUFA11	Ndufa11	NADH dehydrogenase [ubiquinone] 1 alpha subcomplex subunit 11	-0.400389	0
28	**Ndufa13**	NDUFA13	Ndufa13	NADH dehydrogenase [ubiquinone] 1 alpha subcomplex subunit 13	-0.500268	0
29	**Ndufa2**	NDUFA2	Ndufa2	NADH dehydrogenase [ubiquinone] 1 alpha subcomplex subunit 2	-0.645568	0
30	**Ndufa3**	NDUFA3	Ndufa3	NADH dehydrogenase [ubiquinone] 1 alpha subcomplex subunit 3	-0.633872	0
31	**Ndufa4**	NDUFA4	Ndufa4	Cytochrome c oxidase subunit NDUFA4	-0.647713	0

32	**Ndufa5**	NDUFA5	Ndufa5	NADH dehydrogenase [ubiquinone] 1 alpha subcomplex subunit 5	-0.469147	0
33	**Ndufa6**	NDUFA6	Ndufa6	NADH dehydrogenase [ubiquinone] 1 alpha subcomplex subunit 6	-0.687919	0
34	**Ndufa7**	NDUFA7	Ndufa7	NADH dehydrogenase [ubiquinone] 1 alpha subcomplex subunit 7	-0.55122	0
35	**Ndufa8**	NDUFA8	Ndufa8	NADH dehydrogenase [ubiquinone] 1 alpha subcomplex subunit 8	-0.409007	0
36	**Ndufa9**	NDUFA9	Ndufa9	NADH dehydrogenase [ubiquinone] 1 alpha subcomplex subunit 9, mitochondrial	-0.503337	0
37	**Ndufab1**	NDUFAB1	Ndufab1	Acyl carrier protein, mitochondrial	-0.534754	0
38	**Ndufb10**	NDUFB10	Ndufb10	NADH dehydrogenase [ubiquinone] 1 beta subcomplex subunit 10	-0.461899	0

39	**Ndufb2**	NDUFB2	Ndufb2	NADH dehydrogenase [ubiquinone] 1 beta subcomplex subunit 2, mitochondrial	-0.498395	0
40	**Ndufb3**	NDUFB3	Ndufb3	NADH dehydrogenase [ubiquinone] 1 beta subcomplex subunit 3	-0.4932	0
41	**Ndufb4**	NDUFB4	Ndufb4	NADH dehydrogenase [ubiquinone] 1 beta subcomplex subunit 4	-0.581655	0
42	**Ndufb4**	NDUFB4	Ndufb4b	NADH dehydrogenase [ubiquinone] 1 beta subcomplex subunit 4	-0.581655	0
43	**Ndufb5**	NDUFB5	Ndufb5	NADH dehydrogenase [ubiquinone] 1 beta subcomplex subunit 5, mitochondrial	-0.53372	0
44	**Ndufb6**	NDUFB6	Ndufb6	NADH dehydrogenase [ubiquinone] 1 beta subcomplex subunit 6	-0.519488	0
45	**Ndufb7**	NDUFB7	Ndufb7	NADH dehydrogenase [ubiquinone] 1 beta subcomplex subunit 7	-0.374883	0

46	**Ndufb8**	NDUFB8	Ndufb8	NADH dehydrogenase [ubiquinone] 1 beta subcomplex subunit 8, mitochondrial	-0.523241	0
47	**Ndufb9**	NDUFB9	Ndufb9	NADH dehydrogenase [ubiquinone] 1 beta subcomplex subunit 9	-0.530734	0
48	**Ndufc1**	NDUFC1	Ndufc1	NADH dehydrogenase [ubiquinone] 1 subunit C1, mitochondrial	-0.655074	0
49	**Ndufc2**	NDUFC2	Ndufc2	NADH dehydrogenase [ubiquinone] 1 subunit C2	-0.485233	0
50	**Ndufs1**	NDUFS1	Ndufs1	NADH-ubiquinone oxidoreductase 75 kDa subunit, mitochondrial	-0.641851	0
51	**Ndufs2**	NDUFS2	Ndufs2	NADH dehydrogenase [ubiquinone] iron-sulfur protein 2, mitochondrial	-0.57924	0
52	**Ndufs3**	NDUFS3	Ndufs3	NADH dehydrogenase [ubiquinone] iron-sulfur protein 3, mitochondrial	-0.438247	0

53	**Ndufs4**	NDUFS4	Ndufs4	NADH dehydrogenase [ubiquinone] iron-sulfur protein 4, mitochondrial	-0.477776	0
54	**Ndufs5**	NDUFS5	Ndufs5	NADH dehydrogenase [ubiquinone] iron-sulfur protein 5	-0.413575	0
55	**Ndufs6**	NDUFS6	Ndufs6	NADH dehydrogenase [ubiquinone] iron-sulfur protein 6, mitochondrial	-0.567075	0
56	**Ndufs7**	NDUFS7	Ndufs7	NADH dehydrogenase [ubiquinone] iron-sulfur protein 7, mitochondrial	-0.442479	0
57	**Ndufs8**	NDUFS8	Ndufs8	NADH dehydrogenase [ubiquinone] iron-sulfur protein 8, mitochondrial	-0.432103	0
58	**Ndufv1**	NDUFV1	Ndufv1	NADH dehydrogenase [ubiquinone] flavoprotein 1, mitochondrial	-0.613443	0
59	**Ndufv2**	NDUFV2	Ndufv2	NADH dehydrogenase [ubiquinone] flavoprotein 2, mitochondrial	-0.561687	0
60	**Ndufv3**	NDUFV3	Ndufv3	NADH dehydrogenase [ubiquinone] flavoprotein 3, mitochondrial	-0.53266	0

61		O(,2) + 2-Decaprenyl-6-methoxyphenol = H(,2)O + 2-Decaprenyl-6-methoxy-1,4-benzoquinone				
62	**mt-Nd1**	Respiratory Complex I (NADH-ubiquinone oxidoreductase)	ND1		-0.312574	0
63	**mt-Nd2**	Respiratory Complex I (NADH-ubiquinone oxidoreductase)	ND2		-0.345866	0
64	**mt-Nd3**	Respiratory Complex I (NADH-ubiquinone oxidoreductase)	ND3		-0.253271	0
65	**mt-Nd4**	Respiratory Complex I (NADH-ubiquinone oxidoreductase)	ND4		-0.37974	0
66	**mt-Nd4l**	Respiratory Complex I (NADH-ubiquinone oxidoreductase)	ND4L		-0.431259	0
67	**mt-Nd5**	Respiratory Complex I (NADH-ubiquinone oxidoreductase)	ND5		-0.500391	0

68		Respiratory Complex I (NADH-ubiquinone oxidoreductase)	ND6			
69	**Ndufa1**	Respiratory Complex I (NADH-ubiquinone oxidoreductase)	Ndufa1		-0.638382	0
70	**Ndufa10**	Respiratory Complex I (NADH-ubiquinone oxidoreductase)	Ndufa10		-0.526248	0
71	**Ndufa11**	Respiratory Complex I (NADH-ubiquinone oxidoreductase)	Ndufa11b		-0.400389	0
72	**Ndufa12**	Respiratory Complex I (NADH-ubiquinone oxidoreductase)	Ndufa12		-0.490431	0
73	**Ndufa13**	Respiratory Complex I (NADH-ubiquinone oxidoreductase)	Ndufa13		-0.500268	0
74	**Ndufa2**	Respiratory Complex I (NADH-ubiquinone oxidoreductase)	Ndufa2		-0.645568	0
75	**Ndufa3**	Respiratory Complex I (NADH-ubiquinone oxidoreductase)	Ndufa3		-0.633872	0

76	**Ndufa4**	Respiratory Complex I (NADH-ubiquinone oxidoreductase)	Ndufa4		-0.647713	0
77	**Ndufa5**	Respiratory Complex I (NADH-ubiquinone oxidoreductase)	Ndufa5		-0.469147	0
78	**Ndufa6**	Respiratory Complex I (NADH-ubiquinone oxidoreductase)	Ndufa6		-0.687919	0
79	**Ndufa7**	Respiratory Complex I (NADH-ubiquinone oxidoreductase)	Ndufa7		-0.55122	0
80	**Ndufa8**	Respiratory Complex I (NADH-ubiquinone oxidoreductase)	Ndufa8		-0.409007	0
81	**Ndufa9**	Respiratory Complex I (NADH-ubiquinone oxidoreductase)	Ndufa9		-0.503337	0
82	**Ndufab1**	Respiratory Complex I (NADH-ubiquinone oxidoreductase)	Ndufab1		-0.534754	0
83	**Ndufb10**	Respiratory Complex I (NADH-ubiquinone oxidoreductase)	Ndufb10		-0.461899	0

84	**Ndufb11**	Respiratory Complex I (NADH-ubiquinone oxidoreductase)	Ndufb11		-0.53213	0
85	**Ndufb2**	Respiratory Complex I (NADH-ubiquinone oxidoreductase)	Ndufb2		-0.498395	0
86	**Ndufb3**	Respiratory Complex I (NADH-ubiquinone oxidoreductase)	Ndufb3		-0.4932	0
87	**Ndufb4**	Respiratory Complex I (NADH-ubiquinone oxidoreductase)	Ndufb4		-0.581655	0
88	**Ndufb4**	Respiratory Complex I (NADH-ubiquinone oxidoreductase)	Ndufb4b		-0.581655	0
89	**Ndufb5**	Respiratory Complex I (NADH-ubiquinone oxidoreductase)	Ndufb5		-0.53372	0
90	**Ndufb6**	Respiratory Complex I (NADH-ubiquinone oxidoreductase)	Ndufb6		-0.519488	0
91	**Ndufb7**	Respiratory Complex I (NADH-ubiquinone oxidoreductase)	Ndufb7		-0.374883	0

92	**Ndufb8**	Respiratory Complex I (NADH-ubiquinone oxidoreductase)	Ndufb8		-0.523241	0
93	**Ndufb9**	Respiratory Complex I (NADH-ubiquinone oxidoreductase)	Ndufb9		-0.530734	0
94	**Ndufc1**	Respiratory Complex I (NADH-ubiquinone oxidoreductase)	Ndufc1		-0.655074	0
95	**Ndufc2**	Respiratory Complex I (NADH-ubiquinone oxidoreductase)	Ndufc2		-0.485233	0
96	**Ndufs1**	Respiratory Complex I (NADH-ubiquinone oxidoreductase)	Ndufs1		-0.641851	0
97	**Ndufs2**	Respiratory Complex I (NADH-ubiquinone oxidoreductase)	Ndufs2		-0.57924	0
98	**Ndufs3**	Respiratory Complex I (NADH-ubiquinone oxidoreductase)	Ndufs3		-0.438247	0
99	**Ndufs4**	Respiratory Complex I (NADH-ubiquinone oxidoreductase)	Ndufs4		-0.477776	0

100	**Ndufs5**	Respiratory Complex I (NADH-ubiquinone oxidoreductase)	Ndufs5		-0.413575	0
101	**Ndufs6**	Respiratory Complex I (NADH-ubiquinone oxidoreductase)	Ndufs6		-0.567075	0
102	**Ndufs7**	Respiratory Complex I (NADH-ubiquinone oxidoreductase)	Ndufs7		-0.442479	0
103	**Ndufs8**	Respiratory Complex I (NADH-ubiquinone oxidoreductase)	Ndufs8		-0.432103	0
104	**Ndufv1**	Respiratory Complex I (NADH-ubiquinone oxidoreductase)	Ndufv1		-0.613443	0
105	**Ndufv2**	Respiratory Complex I (NADH-ubiquinone oxidoreductase)	Ndufv2		-0.561687	0
106	**Ndufv3**	Respiratory Complex I (NADH-ubiquinone oxidoreductase)	Ndufv3		-0.53266	0

107		S-Adenosyl-L-methionine + 2-Decaprenyl-6-methoxy-1,4-benzoquinone = S-Adenosyl-L-homocysteine + 2-decaprenyl-3-methyl-6-methoxy-1,4-benzoquinone				
108		S-Adenosyl-L-methionine + 3-Decaprenyl-4,5-dihydroxybenzoate = S-Adenosyl-L-homocysteine + 3-Decaprenyl-4-hydroxy-5-methoxybenzoate				
109		S-Adenosyl-L-methionine + demethyl-Coenzyme-Q10 = Coenzyme-Q10 + S-Adenosyl-L-homocysteine				
110		TAT	Tat	Tyrosine aminotransferase		
111		Ubiquinol-10 intracellular anatomical structure		Ubiquinol-10		

112		all-trans-Hexaprenyl diphosphate + 4-Hydroxy-benzoic acid = Pyrophosphate + 3-Hexaprenyl-4-hydroxy-benzoic acid					
113		all-trans-Hexaprenyl diphosphate intracellular anatomical structure		all-trans-Hexaprenyl diphosphate			
114	**Coq2**	coenzyme Q2 homolog, prenyltransferase (yeast)	Coq2	4-hydroxybenzoate polyprenyltransferase	-0.364377	0	
115		demethyl-Coenzyme-Q10 intracellular anatomical structure		demethyl-Coenzyme-Q10			
116		p-Coumaric acid extracellular region		p-Coumaric acid			
117		p-Hydroxyphenyllactic acid intracellular anatomical structure		p-Hydroxyphenyllactic acid			
118		p-Hydroxyphenylpyruvic acid intracellular anatomical structure		p-Hydroxyphenylpyruvic acid			
