# Supplementary material for: Intrinsic TGF-β signaling attenuates proximal tubule mitochondrial injury and inflammation in chronic kidney disease
Source: Nat Commun. 2023 Jun 3;14:3236. doi: 10.1038/s41467-023-39050-y (PMC10239443; doi:10.1038/s41467-023-39050-y)
Supplement: Supplementary file 3 — Reporting Summary [file 41467_2023_39050_MOESM3_ESM.pdf]

nature portfolio

Corresponding author(s): STELLOR NLANDU KHODO

Last updated by author(s): 2023.04.21

Reporting Summary

Nature Portfolio wishes to improve the reproducibility of the work that we publish. This form provides structure for consistency and transparency in reporting. For further information on Nature Portfolio policies, see our Editorial Policies and the Editorial Policy Checklist.

Statistics

For all statistical analyses, confirm that the following items are present in the figure legend, table legend, main text, or Methods section.

n/a	Confirmed	
☐	☒	The exact sample size (*n*) for each experimental group/condition, given as a discrete number and unit of measurement
☐	☒	A statement on whether measurements were taken from distinct samples or whether the same sample was measured repeatedly
☐	☒	The statistical test(s) used AND whether they are one- or two-sided<br>*Only common tests should be described solely by name; describe more complex techniques in the Methods section.*
☒	☐	A description of all covariates tested
☐	☒	A description of any assumptions or corrections, such as tests of normality and adjustment for multiple comparisons
☒	☐	A full description of the statistical parameters including central tendency (e.g. means) or other basic estimates (e.g. regression coefficient) AND variation (e.g. standard deviation) or associated estimates of uncertainty (e.g. confidence intervals)
☐	☒	For null hypothesis testing, the test statistic (e.g. *F*, *t*, *r*) with confidence intervals, effect sizes, degrees of freedom and *P* value noted<br>*Give P values as exact values whenever suitable.*
☒	☐	For Bayesian analysis, information on the choice of priors and Markov chain Monte Carlo settings
☒	☐	For hierarchical and complex designs, identification of the appropriate level for tests and full reporting of outcomes
☒	☐	Estimates of effect sizes (e.g. Cohen's *d*, Pearson's *r*), indicating how they were calculated

Our web collection on statistics for biologists contains articles on many of the points above.

Software and code

Policy information about availability of computer code

Data collection	A BD FACSymphony 5L cell cytometer was used for flow cytometry. Whole kidney fluorescence and brightfield images were acquired on slide (Zeiss Axio Scan.ZI) using Nikon software ZEN 3.1 (blue edition) for visualization and captures. Electron microscopy images were acquired at different magnification using a transmission electron microscope (Tecnai T12 operating at 100keV using a side mount AMT CCD camera). Imaging was performed using a custom-built multiphoton microscope operating in an inverted mode, and powered by a broadband tunable laser (InSight DeepSee Dual Ultrafast Ti:Sapphire, Spectraphysics, Santa Clara, CA, USA). Intravital imaging was performed with an XLPlan N ×25/1.05 water immersion objective (Olympus, Tokyo, Japan) and emitted light was collected through four highly sensitive gallium-arsenide-phosphide photomultiplier tubes (Hamamatsu, Japan) in a non-descanned epifluorescence detection mode. Seahorse (XFp and XFe 96 Analyzers). Western blot images were acquired on Luminescent Image Analyzer (Fujifilm LAS-4000 and VILBER Fusion Fx). Bioluminescence was acquired using Berthold Technologies Centro LB 960 machine. RT-qPCR data was acquired using Agilent (Stratagene Mx3000P and AriaMx) machines. Library construction for spatial transcriptomics was performed on Sensquest Lab Cycler. Sequencing was performed on Illumina Novaseq 6000 (Functional Genomics Center Zurich). We used SpaceRanger from 10X Genomics, Seurat V3 and Seurat v4.1.0, Gimp 2.10.6, GraphPad Prism 9, ImageJ (National Institutes of Health, Bethesda, MD).
Data analysis	RNA Seq reads were mapped using STAR 2.7.0. Gene-level counts generated using Rsubread 2.4.0. Differential expression performed using edgeR, pathway analysis performed using clusterProfiler in R 4.0.3. Differential exon usage performed using JunctionSeq 1.21.0. We used Cloupe brower 6.4.1, Seurat V3 and Seurat v4.1.0, CellchatDB, Metacore and EnrichR (2021) for spatial transcriptomics analyses. All analysis were performed based on the Sushi uzh/sushi: SUSHI: Supporting User for SHell script Integration (github.com) and ezRun uzh/ezRun: An R meta-package for the analysis of Next Generation Sequencing data (github.com). Other used software included R, Cloupe brower 6.4.1, GraphPad Prism 9, ImageJ (1.53), FlowJo (V10.6.1), Wave 2.6. Gimp 2.10.6

For manuscripts utilizing custom algorithms or software that are central to the research but not yet described in published literature, software must be made available to editors and reviewers. We strongly encourage code deposition in a community repository (e.g. GitHub). See the Nature Portfolio guidelines for submitting code & software for further information.

Data

Policy information about availability of data

All manuscripts must include a data availability statement. This statement should provide the following information, where applicable:

- Accession codes, unique identifiers, or web links for publicly available datasets
- A description of any restrictions on data availability
- For clinical datasets or third party data, please ensure that the statement adheres to our policy

RNAseq raw and metadata are available at both GEO accession GSE225545 (https://www.ncbi.nlm.nih.gov/geo/query/acc.cgi?acc=GSE225545) and Zenodo repository (https://zenodo.org/record/7912879#.ZGDp5oRByUl). Visium data and analysis are available at Zenodo repository (https://doi.org/10.5281/zenodo.7635958). Online scRNAseq data used for cell type annotation in Visium were from GSE151658 (https://www.ncbi.nlm.nih.gov/geo/query/acc.cgi?acc=GSE151658)32. snRNA-Seq data were from Zenodo repository under the accession number 4059315 (https://zenodo.org/record/4059315#.ZGDsbYRByUk). Source data are provided with this paper. All other relevant data supporting the key funding of this study are available within the article and its Supplementary information file.

Research involving human participants, their data, or biological material

Policy information about studies with human participants or human data. See also policy information about sex, gender (identity/presentation), and sexual orientation and race, ethnicity and racism.

Reporting on sex and gender	N/A
Reporting on race, ethnicity, or other socially relevant groupings	N/A
Population characteristics	N/A
Recruitment	N/A
Ethics oversight	N/A

Note that full information on the approval of the study protocol must also be provided in the manuscript.

Field-specific reporting

Please select the one below that is the best fit for your research. If you are not sure, read the appropriate sections before making your selection.

☒ Life sciences ☐ Behavioural & social sciences ☐ Ecological, evolutionary & environmental sciences

For a reference copy of the document with all sections, see nature.com/documents/nr-reporting-summary-flat.pdf

Life sciences study design

All studies must disclose on these points even when the disclosure is negative.

Sample size	Based on our preliminary data on renal injury induction where a sample size of 7 animals per group was sufficient to reach statistical significance, and the one hour free consulting with the biostatistician of the office for Animal Welfare and the 3Rs of the University of Zurich (UZH), a calculation of sample size by power analysis using two-way ANOVA (4 groups and 6 comparisons) indicates that we will need 20 mice per group to have 89.2% power to detect a significant difference (alpha equals 0.05) in renal injury between genotypes in the Aristolochic acid and unilateral ureteral obstruction injury models (corrected alpha equals 0.00833 for 6 comparisons; injury score difference of 1.14 and the highest sample standard deviation of 1.286). AA model is known to robustly induce renal injury and statistical significance was reached with lower number of animals. These numbers are calculated/obtained using the UZH sample size online calculator. (http://shiny.math.uzh.ch/git/reinhard.furrer/SampleSizeR/).
Data exclusions	For blood samples, we excluded samples with lower volume than what is required for the analysis and sample with apparent hemolysis. For spatial transcriptomics low quality clusters based on transcript counts, mitochondrial and ribosomal transcript contents were removed.
Replication	All attempts at replication were successful. Experiments were repeated at least three times as reported in the figure legends with statistical significance. Independent biological replicates were run together or in different experiments. Since we used conditionally immortalized PT cells, some experiments were repeated more than three times to improve the statistical significance.
Randomization	After genotyping, floxed (WT) and conditional knockout (KO) animals were allocated into experimental groups by a blinded animal caretaker. WT and KO animals were given special codes, mixed in different cages and randomly assigned in cages to give each animal the same chance of being injured or control.
Blinding	WT and KO animals were given special codes and the genotypes were defined at the end of data analyses. Analyses were performed by

Blinding	experimenter who ignored animal genotype and injury time points. We used animal codes for quantification and determined their genoytypes after the quantification.

Reporting for specific materials, systems and methods

We require information from authors about some types of materials, experimental systems and methods used in many studies. Here, indicate whether each material, system or method listed is relevant to your study. If you are not sure if a list item applies to your research, read the appropriate section before selecting a response.

Materials & experimental systems

n/a	Involved in the study
☐	☒ Antibodies
☐	☒ Eukaryotic cell lines
☒	☐ Palaeontology and archaeology
☐	☒ Animals and other organisms
☒	☐ Clinical data
☒	☐ Dual use research of concern
☒	☐ Plants

Methods

n/a	Involved in the study
☒	☐ ChIP-seq
☐	☒ Flow cytometry
☒	☐ MRI-based neuroimaging

Antibodies

Antibodies used	Antibody's information is listed in the supplementary table 1 and 2. Pgc1a (Merck, Cat. number ST1202, clone 4c1.3, dilution 1:500), CD3 (Abcam, Cat. number ab16669, clone SP7, dilution 1:150), pSMAD 3 (Rockland, Cat. number, dilution 1:500), F4/80 (Abcam, Cat. number ab6640, clone CI:A3-1, dilution 1:150), Kim-1 (R&D Systems, Cat. number AF1817, dilution 1:200), LTL (Vector Laboratories, Cat. number FL-1321, dilution 1:400), Pink1 (Novus, Cat. number BC100-494, dilution 1:1000), LC3A (Cell Signaling, Cat. number 4599S, clone D50G8, dilution 1: 500), OXPHOS (Abcam, Cat. number ab110413, anti-NDUFB8 clone 20E9DH10C12, anti-SDHB clone 21A11AE7, anti-UQCRC2 clone 13G12AF12BB11, anti-MTCO1 clone 1D6E1A8, anti-ATP5A clone 15H4C4, dilution 1:1000), Polg (Santa Cruz, Cat. number sc-390634, clone G-6, dilution 1:200), Tom20 (Santa Cruz, Cat. number sc-11415, dilution 1:1000), alpha-Tubulin (Gentex, Cat. number GTX628802-01, clone GT114, dilution 1:4000), beta-Actin (Sigma, Cat. number A5441, clone AC-15, dilution 1:20000).
Validation	Pgc1a antibodies recognized two bands close to the expected molecular weight, we validated the antibodies using siRNA targeting Pgc1a. Other antibodies recognized antigens at the expected molecular weights and expression patterns.

Eukaryotic cell lines

Policy information about cell lines and Sex and Gender in Research

Cell line source(s)	Proximal tubule (PT) cells derived from male immorto;Tgfbr2floxed mice treated or not with adeno-CRE. PT cells were isolated from male mice and grown at 33°C in DMEM/F12 supplemented with 2.5% fetal bovine serum, hydrocortisone, insulin, transferrin, selenium, triiodothyronine, and penicillin/streptomycin (complete PT media) with IFNg. Prior to experiments, PT cells were moved to 37°C and IFNg removed to induce differentiation. Deletion of TbRII in PT was achieved by adeno-Cre treatment in vitro and verified by immunoblotting and RNAseq.
Authentication	Knockout cells were confirmed by Tgfbr2 expression and response to TGFb1 stimulation.
Mycoplasma contamination	Cells were tested negative for Mycoplasma contamination
Commonly misidentified lines (See ICLAC register)	N/A

Animals and other research organisms

Policy information about studies involving animals; ARRIVE guidelines recommended for reporting animal research, and Sex and Gender in Research

Laboratory animals	Pure FVB mice of 8-12 weeks age
Wild animals	No wild animals were used in this study.
Reporting on sex	These findings apply to male mice. In order to follow up, our previous study, a gender based study was out of the scope of this study and will be foreseen in the upcoming study.
Field-collected samples	The study did not involve samples collected from the field.
Ethics oversight	All procedures were approved by the veterinary office of the canton Zurich, Switzerland (ZH123/19). Prior to experimental

Ethics oversight

commitment, mice were tagged using ear notching in accordance with the Laboratory Animal Services Center (LASC) license 101, and generated tissue were used for genotyping. After genotyping, animals were transferred to the experimental room, where they were allowed to acclimate for at least 7 days before starting experiments. During the acclimation period, mice were randomly assigned as controls or CKD and monitored to ensure water and food ad libitum accessibility every other day. Given the pathocentric strain of CKD models, the use of painkillers is mandatory to minimize procedure-related pain. Mice were monitored every other day during 2 weeks of intraperitoneal AA injections (six injections every other day), then every day in the acute phase (7 days after the last AA injection) and finally every other day afterward until the experiment endpoint. Mice were scored for signs of pain (hunched posture, poor grooming, reduced mobility and subsequent body weight loss) every day in the acute phase. According to of our pain scoring criteria, mice were provided wet food pellets and/or administered pre-warmed Ringer's lactate/5% glucose solution and/or buprenorphine (0.1 mg/kg) diluted in 0.9% NaCl (1 ml of 0.3 mg/ml of buprenorphine in 5 ml of 0.9% NaCl to have 2 microliters/g body weight). Buprenorphine is an opioid and strong analgesic that we preferred in this study because of its long lasting effect (6-8 h), and compared to other opioids (butorphanol for instance), it reportedly has minimal hemodynamic side effects which is a very important aspect in this study. Euthanasia was considered in case of failure of pain mitigating measures and at the experimental endpoint. If euthanasia is needed before the experimental endpoint, mice were submitted to 70% CO2 and only the kidneys were collected for further investigation. In case we cannot decrease animal distress in the assigned time period, concerned animal was immediately euthanized and the kidneys were collected for further investigation. At the experiment end point, mice were anesthetized by inhalation of 5% isoflurane in oxygen as carrier gas using the VetFlo stand. After confirmation of complete anesthesia by checking the pedal withdrawal reflex and tail pinch three times, mice were euthanized by cervical dislocation. The personal phone number of the study director and the experimenter including their designed substitutes were purposely put on the animal ID card in order to be contacted for emergency intervention to avoid animals suffering.

Note that full information on the approval of the study protocol must also be provided in the manuscript.

Flow Cytometry

Plots

Confirm that:

- [x] The axis labels state the marker and fluorochrome used (e.g. CD4-FITC).
- [x] The axis scales are clearly visible. Include numbers along axes only for bottom left plot of group (a 'group' is an analysis of identical markers).
- [x] All plots are contour plots with outliers or pseudocolor plots.
- [x] A numerical value for number of cells or percentage (with statistics) is provided.

Methodology

Sample preparation

Kidneys were collected from uninjured and injured animals. After capsule removal, kidneys were immediately placed in ice-cold FACS buffer (2% FBS in PBS), mechanically dissected and mixed with IMDM media containing collagenase IV (600U/ml, Worthington) for 1 hour at 37 oC in a shaker (150 rpm). Single cells were obtained by passing the digested kidneys through 70 µm cell strainers. Samples were washed once with FACS buffer and treated with ACK lysis buffer (150 mM NH4Cl, 10 mM KHCO3 and 0.1 mM Na2EDTA in dH2O pH 7.2) for 1 min to get rid of red blood cells. Afterwards, samples were washed with FACS buffer and re-suspended in 8 ml 40% (w/v) percoll gradient solution, 70% percoll was underplayed and centrifuged for 30 min at 860 x g at room temperature. After centrifugation 5-6 ml percoll gradient was removed and the leukocyte ring at the inter-phase transferred to a new tube for washing with FACS buffer. Samples were divided into 3 groups for extracellular myeloid, extracellular lymphoid and intracellular staining.

Instrument

FACSymphony 5L cell cytometer was used for acquisition

Software

FlowJo for further analysis.

Cell population abundance

After mechanical and enzymatic digestion, the single cells were created and epithelial cells, red blood cells and immune cells were further separated using Percoll gradient separation. The yield of immune cells is always based on the step after Percoll gradient separation. Immune cells are further purified from red blood cells by a short exposure to ACK lysis buffer. The cell number, as well as the percentage of each immune cell population is defined using certain markers notably CD45. Abundance of relevant cell populations can be visually seen in the gating strategy, as well as in the excel sheets provided for each cell population; myeloid, lymphoid and cytokine producing cells.

Gating strategy

Single cells were preliminary selected in terms of size (i.e to avoid doublets) with FSC/SSC gating. Singlets were further selected using SSC-H gating. Interested population of cells supposed to be analyzed with alive cells. Therefore, a Live-dead Flow Cytometry marker used to selected alive cells in our analysis. Thereafter, specific markers used to gate and analyze for each immune cell type as stated in the gating strategy and antibody list we have provided in supplementary material.

- [x] Tick this box to confirm that a figure exemplifying the gating strategy is provided in the Supplementary Information.
